# The ubiquity and ancestry of insect *doublesex*

**DOI:** 10.1038/srep13068

**Published:** 2015-08-17

**Authors:** Dana C. Price, Andrea Egizi, Dina M. Fonseca

**Affiliations:** 1 Rutgers University, Department of Entomology, New Brunswick, NJ, USA; 2 Graduate Program in Ecology and Evolution, Rutgers University, New Brunswick, NJ, USA

## Abstract

The *doublesex* (*dsx*) gene functions as a molecular switch at the base of the insect sex determination cascade, and triggers male or female somatic sexual differentiation in *Drosophila*. Having been reported from only seven current insect orders, the exact phylogenetic distribution of *dsx* within the largest Arthropod sub-phylum, the Hexapoda, is unknown. To understand the evolution of this integral gene relative to other arthropods, we tested for the presence of *dsx* within public EST and genome sequencing projects representative of all 32 hexapod orders. We find the *dsx* gene to be ubiquitous, with putative orthologs recovered from 30 orders. Additionally, we recovered both alternatively spliced and putative paralogous *dsx* transcripts from several orders of hexapods, including basal lineages, indicating the likely presence of these characteristics in the hexapod common ancestor. Of note, other arthropods such as chelicerates and crustaceans express two *dsx* genes, both of which are shown to lack alternative splicing. Furthermore, we discovered a large degree of length heterogeneity in the common region of *dsx* coding sequences within and among orders, possibly resulting from lineage-specific selective pressures inherent to each taxon. Our work serves as a valuable resource for understanding the evolution of sex determination in insects.

Somatic sexual differentiation is fundamentally conserved among metazoans. The development of distinct male and female phenotypes integral to sexual reproduction is directed by the *Doublesex*/Mab-3 Related Transcription factor (DMRT) family of zinc-finger proteins^1^,^2^. The DMRT gene family has been described from most animal genomes studied thus far, and likely has pre-eumetazoan origins^3^. Despite conservation of DMRT presence, the specific manner in which members of the family act upon the multigene cascade governing sexual development varies widely among animal phyla^4^,^5^,^6^. One of the most functionally characterized DMRT genes is insect *doublesex*, the gene that acts as the terminal “double-switch” in the insect sex determination cascade.

Best elucidated in the model fly *Drosophila melanogaster*^7^,^8^, the insect sex-determination molecular cascade consists of a primary signal (e.g X:A ratio, W/Y chromosomes, male-determining loci; see references^9^,^10^,^11^) that facilitates sex-specific splicing of intermediary factors such as *sexlethal (sxl)* and *transformer* (*tra*). Functional SXL^F^ protein (produced only in the female) then splices *tra* pre-mRNA to encode functional TRA^F^ protein, which in turn splices *dsx* pre-mRNA in the female specific manner. Male-specific SXL^M^ and TRA^M^ are truncated and do not appear functional, resulting in male-specific *dsx* mRNA. As *sxl* is limited to *Drosophila* and *tra* appears lost in several insect lineages^12^,^13^,^14^, additional intermediary factors may yet remain undiscovered. The genes targeted by female-specific DSX^F^ and male-specific DSX^M^ transcription factors remain elusive, however recent work has shown that *D. melanogaster* DSX binds thousands of targets from multiple tissues in both sexes^15^. Many of these targets overlap with those identified for mouse DMRT1, attesting to the conserved functional nature of eukaryote sex determination. In addition to determining sexual fate, *dsx* is also known to influence the development of both behavioral and morphological secondary sex characteristics^16^,^17^,^18^ and therefore plays several key roles in determining the specific sexual characteristics of a species.

DSX proteins retain conserved domains essential for transcription factor activity and oligomerization: an atypical zinc-finger DNA-binding DM domain (herein OD1) conserved throughout the DMRT gene family that exhibits a characteristic C2H2C4 Cys-His configuration^19^,^20^,^21^, and a dimerization domain (herein OD2) specific to *doublesex*^22^. The OD2 domain is primarily responsible for enhancing dimerization strength and thus OD1-DNA recognition^23^, a function achieved via ubiquitin-associated domain folds^24^. Mutations blocking dimer formation have produced intersex individuals^25^, thus demonstrating the conformation is integral to protein function.

*Dsx* transcripts have been recovered in the crustacean *Daphnia magna*^26^, as well as the arachnid *Metaseiulus occidentalis*^27^, both of which are hexapod outgroups within the Arthropoda. In these organisms, *dsx* pre-mRNA shows no sign of alternative splicing and directs sexual fate via sex-biased expression in males. The extent of conservation of this critical gene throughout hexapods however remains unknown, as thus far *dsx* homologs have been recovered from genome and/or transcriptome data of only seven insect orders (see^14^). As the development of genetic sexing strains of insect vectors is a major focus of sterile insect technique (SIT, a method of population control for disease vectors and other pest species^28^), genes that influence sexual development such as *dsx* are thus optimal targets for molecular manipulation of sex ratios^29^.

To understand the breadth and functional conservation of these genes throughout insects (as well as other Metazoa) we used public transcriptome datasets, including recent EST data generated for all 32 currently recognized hexapod orders^30^ to qualify the presence of *dsx* in this diverse group. We report putative *dsx* orthologs (as evidenced by recovery of an OD2-encoding EST) from 30 orders, 22 of which were represented by at least one EST encoding both an OD1 and downstream OD2 domain. Included in this latter group were species of primitive entognathous hexapods (i.e. Protura) and the derived pterygote insects, confirming the presence of *doublesex* in the common ancestor of the Hexapoda^26^.

## Results and Discussion

### Sequence and phylogenetic conservation of hexapod *doublesex*

**Figure 1:**
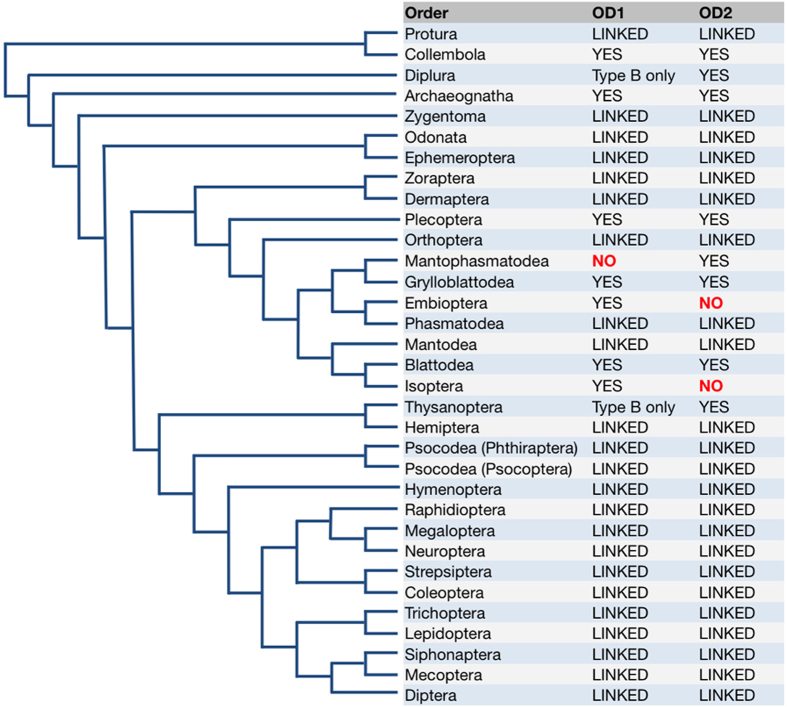
Summary of evidence for hexapod *doublesex* recovered in this study. Orders for which we identified a high-confidence *dsx* EST encoding both OD1 and OD2 domains (see Fig. S1) are marked ‘LINKED’; those for which the domain was found on a singleton EST contig are marked “YES” (see Figs S2, S3 and S4), and those for which we recovered only a Type-B OD1 domain (i.e Type-A absent, see Fig. S3) are annotated as such. Phylogeny as per Misof *et al*. (2014).

We recovered *doublesex* EST contigs encoding both an OD1 and OD2 domain (designated herein as “high-confidence” *dsx* EST contigs) from 22 hexapod orders (Fig. 1, Supplementary Fig. S1 online). We also recovered evidence for both domains existing as singleton (or encoded on separate) contigs in an additional 7 orders (See Fig. 1 for summary; Supplementary Figs S2, S3, S4 online contain all singletons from the analysis). We failed to identify contigs encoding one of the two domains from the remaining three orders: we found no evidence for the OD2 domain from the Embiopteran and Isopteran data, nor OD1 from the Mantophasmatodea. In addition to the EST contigs deposited in NCBI, we assembled *de novo* contigs from short read RNAseq libraries (see Methods) for *Sipyloidea sipylus* (Phasmatodea) and *Lepismachilis y-signata* (Archaeognatha) that contained high-confidence ESTs.

With the exception of four orders, all high-confidence *doublesex* ESTs recovered maintained a conserved Lysine amino acid residue at position 14 of the DM/OD1 domain alignment, most frequently with a Thr-Pro-Pro-Asn-like motif at positions 1–4 (Supplementary Figs S1, S2 online; herein referred to as a type-A OD1 motif). There were multiple instances, however, where we identified ESTs encoding an OD1 domain (often from species for which we had already identified a type-A OD1-encoding EST) with an Ile-Ser-Cys beginning at position 14 and an Arg-Thr-Pro-Lys-like motif at positions 1–4 (Supplementary Fig. S3 online; herein a type-B OD1 motif). As the type-B OD1 motif was not present in any high-confidence *dsx* transcript (members of the Zoraptera and Hemiptera contained proximal matches), we find type-B singletons alone to be poor evidence for the presence of insect *dsx* and may represent a convergent zinc-finger domain from an alternative DMRT-superfamily gene (or alternately, a paralog with highly diverged or absent OD2 domain). Of note, the conserved lysine at position 21 responsible for maintaining a salt bridge with the target DNA molecule^31^ was maintained in all putative OD1 domain sequences of A and B types.

We failed to recover conclusive evidence for the presence of *doublesex* from the termite (Isoptera) data, as only a single type-A OD1 motif was recovered with no evidence for OD2. Two BLAST hits to a type-B-like OD1 motif (contigs Znev_05388 and Znev_16235) were recorded from the *Zootermopsis nevadensis* predicted proteome^32^, however these proteins encode a CUE-like (coupling of ubiquitin to ER degradation) DMA domain in the C-terminus (cd14370, Pfam family PF03474) characteristic of the DMRT family but not of *doublesex*. No BLAST hits to OD2 were reported from *Z. nevadensis*. A profile HMM search using the model we created (see Methods) failed to identity an OD2 motif. As a final check, we searched the *Nasutitermes takasagoensis* raw sequencing reads generated by Hayashi *et al*.^33^ in NCBI SRA accession DRR013047 again using TBLASTN and profile HMM. This returned a single read (G5ZWOJF02FLJ2Z; Suplementary Fig. S3) encoding a type B-like OD1 motif, with no evidence for OD2.

Similarly, we did not recover an OD2 domain from assembled Embiopteran (*Haploembia palaui* and *Aposthonia japonica)* transcriptome data, thus we scanned their constitutive raw short-read RNAseq libraries (of Misof *et al*.^30^) via TBLASTN and profile HMM as described previously. No OD2 hits were recovered in either test from either library. The *H. palaui* EST contig that was found to encode a type-A OD1 (Supplementary Fig. S2 online) encodes a stop codon 423bp downstream of the domain, thus terminating the putative *dsx* protein without evidence of a recognizable OD2 motif. As we cannot rule out *dsx* expression/library preparation biases and sequence divergence for these results, it is likely that further transcriptome and particularly genomic sequence will be required to identify and describe the *dsx* locus and mRNA splicing within the Isoptera and Embioptera. These additional data will confirm whether the dimerization domain has indeed been lost or instead has diverged significantly (beyond our current ability to identify) from the other insects. Loss of the dimerization (OD2) domain would almost certainly impact the DNA-binding affinity of DSX^23^, however protein function is not lost entirely as DM monomers have been shown to form a low affinity complex in the DNA minor groove and induce DNA-dependent folding^21^.

Since we recovered only singleton OD1 and OD2 domains from the roaches (Blattodea), we chose to query the assembled genome of the German cockroach *Blattella germanica* in an attempt to isolate a genomic contig encoding both OD1 and OD2 domains that could be considered high-confidence. A single type-A OD1 motif was recovered on contig JPZV01136765, while a single OD2 domain motif was identified on positions 3574–3717 of contig JPZV01136787 (Supplementary Figs S2, S3 online). Three additional type-B OD1 motifs were recovered, however no hits shared a common genomic contig and thus we were unable to link them. The genome contig encoding the OD1 domain contains 3.6 kb of sequence downstream of the domain, and the contig encoding the OD2 domain contains 3.5 kb of upstream sequence; this is well within the range of intron sizes present between the two domains in dipteran *dsx* gene models^34^,^35^,^36^, thus it remains possible the two domains are linked and were recovered on separate contigs of a fragmented assembly.

Several taxa were found to possess modified zinc finger structures responsible for binding target DNA and modulating transcription. The canonical C2H2C4 Cys-His motif^19^ was notably modified in the high-confidence structures of the Orthoptera (C2H2CHC2) and Zoraptera (C2H3C3) (Supplementary Fig. S1 online). Additional cysteine and histidine residues were observed in members of multiple orders that may affect zinc ion coordination (e.g *Metallyticus splendidus*, *Mantis religiosa*, *Corydalus cornutus, Xenophysella greensladeae*), however further protein biochemistry will be required to qualify the exact effects on DNA-binding affinity.

### Gene copy number

The nearest hexapod outgroup taxa with reported *doublesex* homologs are the water flea *Daphnia magna* (Arthropoda: Crustacea: Branchiopoda: Cladocera: Daphniidae)^26^ and the predatory mite *Metaseiulus occidentalis* (Arthropoda: Chelicerata: Acari: Phytoseiidae)^27^. Both organisms express two copies of *dsx* and lack sex-specific alternative splicing in favor of sex-biased expression in males. The possibility thus exists that the hexapod common ancestor may have encoded two or more *dsx* genes.

Our analyses recovered at least one high-confidence *dsx* EST (encoding both OD1 and OD2 domains) and either an additional divergent OD2-encoding singleton or a second high-confidence EST (i.e. putative evidence for *dsx* paralogy) from eight hexapod orders: Protura, Zygentoma, Ephemeroptera, Zoraptera, Phasmatodea, Mantodea, Hymenoptera and Diptera (all examples illustrated in Supplementary Fig. S1). In all three species of Zygentoma reported here, we recovered both a high-confidence *dsx* EST and a second OD2-encoding singleton. The amino acid sequence of the high-confidence contig in each case was found to diverge from the “consensus” OD2 amino acid sequence to a greater degree (e.g lack of double-Leucine and Valine residues in the N-terminal direction and downstream glutamic acid) than did the singleton, and produced higher domain e-values in homology searches against the profile HMM. Expression of the diverged transcript also appears to be much higher than that of the canonical form; the zygentoman libraries SRR921654, SRR921648 and SRR921568 (*Tricholepidion gertschi*, *Atelura formicaria* and *A. formicaria*, respectively) contained 148, 114 and 164 respective reads that mapped to the OD2 domain of the singleton EST, while 2, 2 and 5 reads from the same libraries mapped to the OD2 domain on the high-confidence EST (not shown). Interestingly, high-confidence ESTs identified within the Zygentoman data exhibit a greater degree of amino acid similarity to the high-confidence ESTs identified within the Ephemeroptera than to their conspecific singleton reads encoding an OD2 domain (illustrated in Supplementary Fig. S5 online). In turn, the singleton reads encoding an OD2 domain are more similar between the two orders than within, indicating these transcripts likely represent a *dsx* gene duplication in a common ancestor of the two orders that sorted accordingly.

The Zoraptera appear to maintain two copies of *dsx*, both of which were recovered as high-confidence ESTs from *Zorotypus caudelli* and *Zorotypus gurneyi* (Supplementary Fig. S1 online). One copy exhibits a C2H2C4 zinc finger motif (a hallmark of the DMRT family, see Introduction) while the other has lost a conserved cysteine to form a modified C2H2C3 finger and encodes a diverged OD2 domain. A third high-confidence EST was recovered from *Zorotypus caudelli* that contained a modification to the zinc finger as above, yet differed in amino acid sequence.

Although previous studies have reported *dsx* in single-copy from the derived orders of holometabolous insects^34^,^37^, we find evidence for duplication in single members of the Hymenoptera (*Sphaeropthalma orestes OD1* = *62%, OD2* = *63%*), Coleoptera (*Meloe violaceus*) and Diptera (*Belgica antarctica*). Further taxon sampling will be required to determine whether these represent individual cases of gene duplication, or retention of an ancestral state. Additionally, multiple members of the Coleoptera (*Gyrinus marinus, Meligethes aeneus* and *Onthophagus nigriventris)* were found to encode a second OD2 domain downstream of the first (Supplementary Figs S1, S4 online). The duplicate domain recovered from *Meligethes aeneus* overlaps a portion of the upstream OD2 and begins at the first amino acid located downstream of the splice donor involved in alternative splicing of other coleopteran *dsx*^37^. The remaining examples from *G. marinus* and *O. nigriventris* encode the second domain 425nt and 30nt respectively downstream of the first, however both are in a different reading frame. In the case of *Meloe violaceous*, we recovered a high-confidence *dsx* EST and an additional singleton OD2 contig that encodes the domain downstream of a stop codon. In the absence of sequencing or assembly artifacts, these data suggest that the duplicated OD2 domains are not translated, yet have undergone domain duplication and/or gene elongation at some point in the evolutionary history of beetles.

### Alternative splicing

**Figure 2:**
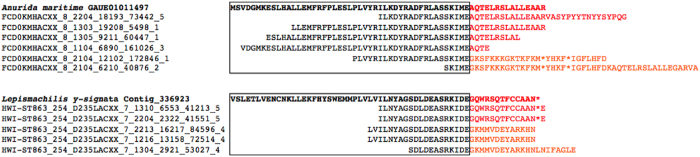
Alternative splicing of *doublesex* transcripts from *Anurida maritime* (Collembola) and *Lepismachilis y-signata* (Archaeognatha). The consensus EST contig from each species (top, bold text), with short-read data mapped below illustrates the diverging transcript isoforms. The common 5′ OD2 sequence is boxed in each species, while the diverging 3′ ends are in red and orange text.

Insect male and female-specific sexual development has been shown in multiple species to proceed via sex-specific splicing of *doublesex* pre-mRNA^7^,^34^,^35^,^37^,^38^,^39^,^40^. The closest insect outgroups for which a *dsx* homolog has been recovered are the branchiopod crustacean genus *Daphnia* and the chelicerate *Metaseiulus occidentalis*, both of which regulate sexual dimorphism via transcript abundance with no evidence of sex-specific alternative splicing^26^,^41^. To assess the extent of *dsx* alternative (and potentially sexually dimorphic) splicing within the hexapods (currently reported only from within the holometabolous insects), we mapped raw sequencing reads (retrieved from the NCBI SRA and corresponding to the transcriptome assemblies used in this paper) from basal orders (Protura, Collembola, Diplura, Archaeognatha and Zygentoma) to the 3′ end of the respective OD2 domain identified for each. We chose this location as it has been shown to harbor the sex-specific exon in the Diptera^34^,^35^,^42^,^43^ and Coleoptera^37^. We identified multiple reads from both the Archaeognatha (*Lepismachilis y-signata*) and Collembola (*Anurida maritima*) that support an alternative *dsx* isoform that diverges immediately downstream of the Glutamic acid residue encoded in the 3′ OD2 mRNA (Fig. 2). This site is identical to that from which alternative splicing occurs in examples from the holometabola (see above references). These results suggest that not only *dsx* but also its alternative splicing were present in the hexapod common ancestor. Further work (i.e. RT-PCR) will be required to confirm these alternate isoforms, and to qualify their sex-specific expression.

### Evolution of *doublesex*

**Figure 3:**
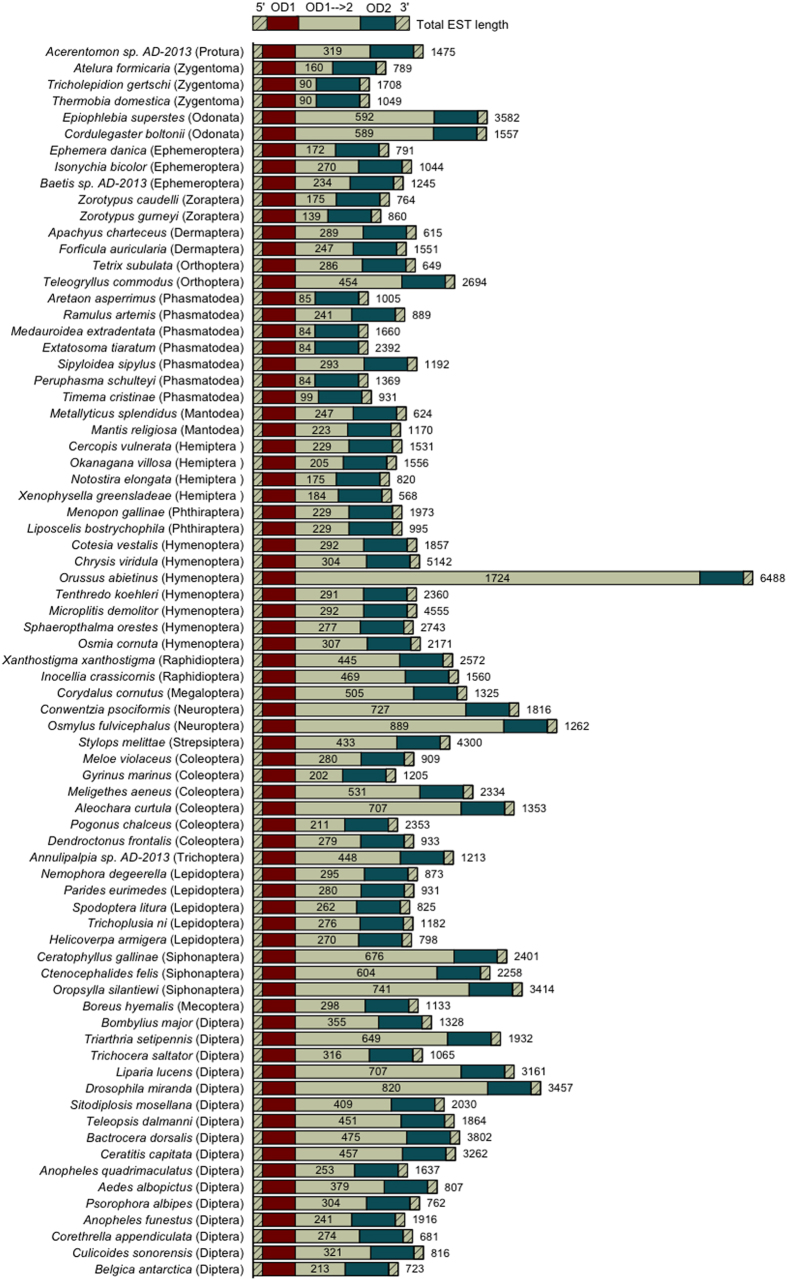
Distance in nucleotides (grey) between the OD1 (red) and OD2 domains (blue), for each high-confidence *doublesex* transcript reported in this study. Total EST length (which may include untranslated regions) is to the right of each bar.

We observed significant length heterogeneity between *doublesex* homologs. The length (in nucleotides) between the OD1 and OD2 domains varied widely both within and between orders (Fig. 3). The average distance among all taxa, excluding putative alternate isoforms, was 356 ± 243 bp. The parasitic hymenopteran *Orussus abietinus* exhibited the largest *dsx* fragment recovered at 6,488 bp with an OD1-to-OD2 distance of 1,724 bp (more than 6-fold greater than the other hymenopteran *dsx* ESTs recovered). Whether this pattern is maintained throughout the Orussidae is of future interest, as a second member of the “Symphyta” (*Tenthredo koehleri*) had a much shorter inter-domain distance. The phasmid *Aretaon asperrimus* had the smallest distance bridging the two domains at only 84bp. Signs of positive selection have been reported within the common region of DSX among closely related members of the *Anastrepha fraterculus* species group^44^, and amino acid substitutions upstream of OD1 and between OD1 and OD2 have been associated with markedly different secondary and tertiary protein structures and associated pleiotropic effects in *Papilio*^45^. Additional studies have supported a role of positive selection on the male-specific protein region, implicated in a system of “runaway evolution” due to its developmental influence on secondary sex characteristics and the response of female preference to genetic drift^46^. As the upstream regulators of *dsx* are diverse and differ even among closely related taxa^34^,^35^,^36^, the large variation in gene length reported here may be a product of evolution under lineage-specific sexual selective pressures balancing the above structural, phenotypic and regulatory factors. The degree of length heterogeneity in the common region makes homologous codon identification, saturation estimates and alignment extremely difficult even between congeners, and thus current formal tests for positive selection will be unable to test this hypothesis. Alternative processes such as genetic drift under neutral mutation can thus not be discounted.

## Conclusions

Our results show *doublesex* to be present throughout the Hexapoda, and likely present in the last common ancestor of the subphylum. The recovery of EST contigs that encode both the DNA-binding (DBD/OD1) and dimerization domains (OD2) from members of the primitive Entognatha to the derived orders of holometabolous insects attests to the conservation of this integral double-switch gene in the sex-determination cascade. Of note, we were unable to recover evidence for the OD2 domain in the embiopteran and isopteran datasets. Despite the use of alternative short-read libraries in addition to transcriptome sequence archives, the domain (if present) has diverged beyond our ability to isolate it via profile hidden Markov model and/or BLAST in these orders.

We present evidence that *dsx* likely existed as a paralogous gene in the hexapod common ancestor, as at least two copies are transcribed in the basal Entognatha (among other orders), and also present in sequenced crustacean and chelicerate outgroups. Additionally, the phenomenon of sex-specific alternative splicing, currently undescribed outside of the insects, appears to occur in the most basal orders and is thus likely to have been present in the proto-hexapod. The advent of sex-specific alternative splicing coupled with mutation and eventual loss of *dsx* gene copy in the higher insect orders are perhaps concomitant, with neo-functionalization of the alternate isoform replacing the lost copy. Tandem duplications of the OD2 domain in the Coleoptera reported here, perhaps once functional, appear to have been inactivated via accrual of mutations. The extreme variation in coding sequence length and conservation observed among and within orders may provide evidence of a punctuated molecular response to sexual selection pressures influenced by the phenotypes that *dsx* modulates. The transcripts identified here serve as initial candidates for studying pan-insect evolution, and for development of transgenic sexing techniques for insect pests^28^.

## Methods

We queried the NCBI Transcriptome Shotgun Assembly (TSA), Whole-Genome Shotgun (WGS), and non-redundant (nr) databases via tBLASTn/BLASTp (e-val = 1.0) with the Pfam seed alignments for pfam00751 (DM DNA binding domain [OD1]) and pfam08828 (*Doublesex* dimerization domain [OD2]). Contigs with both domains present were extracted and translated in the proper reading frame as reported by BLAST. We then extracted and manually aligned the two protein domain motifs with their respective orthologs from other orders. These data compose the ‘high-confidence’ dataset, as the presence of both domains on a single EST is strong evidence for *doublesex* homology. We entered the remaining EST contigs encoding a singular OD1 or OD2 domain into the alignment in the same manner. To search for remote (or diverged) homologs, an alignment of OD2 domain sequences (OD1 maintained strong sequence similarity) from the high-confidence dataset was used to construct a profile hidden Markov model (HMM) using HMMER v3.1b1^47^. This profile HMM was then used to query local copies of the above NCBI databases. We examined manually the resultant hits scoring below the inclusion threshold e-value of 0.01, and added candidate ESTs representing orders not recovered in the BLAST analysis to the dataset. Contigs encoding both an OD1 and OD2 domain were added to the high-confidence set.

We used several data sources outside of the NCBI TSA and nr databases as secondary sources of evidence for orders in which we failed to recover a high-confidence *dsx* EST. The *Lepismachilis y-signata* (Archaeognatha) Illumina-generated RNAseq data deposited in NCBI SRA accessions ERR424579, ERR392013, ERR392014, and ERR392008 were downloaded and assembled using the CLC Genomics Workbench de-novo assembler (CLC Bio, Aarhus, DK). A BLAST database was created from the resultant contigs and queried with the OD1 and OD2 consensus sequences. Both domains were recovered on assembled contigs 383881 and 336923, respectively (Supplementary Fig. S6 online). To ensure our inability to recover an OD2 domain from the Embioptera was not an assembly artifact (i.e. that ‘singleton’ unassembled transcriptome reads may contain a domain hit) we retrieved and queried the *Haploembia palaui* and *Aposthonia japonica* short-read RNAseq libraries (NCBI accessions SRR921605 and SRR921566, respectively) of Misof *et al*.^30^ via TBLASTN and profile HMM as described previously.

The predicted proteome of the dampwood termite *Zootermopsis nevadensis*^32^ was retrieved from http://termitegenome.org and queried via BLASTP with the OD1/2 protein sequences reported here for the roaches (Blattodea, the sister taxon to the termites^30^), and with profile HMM as described previously. As a final check, we searched the *Nasutitermes takasagoensis* raw sequencing reads generated by Hayashi *et al*.^33^ in NCBI SRA accession DRR013047 again using TBLASTN and profile HMM. This process was repeated using the genome contigs of the German cockroach *Blattella germanica* (Blattodea; Baylor College of Medicine Human Genome Sequencing Center [https://www.hgsc.bcm.edu/arthropods/german-cockroach-genome-project]) in an attempt to isolate a high-confidence genomic contig encoding both OD1 and OD2 domains. We queried the assembled genome scaffolds via TBLASTN with roach OD1/2 sequences identified within the NCBI TSA, and with the OD2 profile HMM.

To search for alternative splicing of *dsx* transcripts within the basal hexapods, we retrieved the sequencing reads from the NCBI SRA corresponding to species for which we had recovered an OD2 domain: Protura (SRR921562 [*Acerentomon sp. AD-2013*]), Collembola (SRR921564 [*Anurida maritime*], SRR921647 [*Tetrodontophora bielanensis*], SRR921641 [*Sminthurus viridis*], SRR921635 [*Pogonognathellus sp. AD-2013*]), Diplura (SRR921624 [*Occasjapyx japonicas*]), Archaeognatha (ERR424579, ERR392013, ERR392014, ERR392008 [*Lepismachilis y-signata*], SRR921617 [*Meinertellus cundinamarcensis*]) and Zygentoma (SRR921568 [*Atelura formicaria*], SRR921648 [*Thermobia domestica*], SRR921654 [*Tricholepidion gertschi*]). These reads were six-frame translated and pattern matched to the 3′ end of the OD2 domain to discern divergent splice forms.

## Additional Information

**How to cite this article**: Price, D. C. *et al*. The ubiquity and ancestry of insect *doublesex*. *Sci. Rep*. **5**, 13068; doi: 10.1038/srep13068 (2015).

## Supplementary Material

Supplementary Information
