# Supplementary material for: The ubiquity and ancestry of insect doublesex
Source: Sci Rep. 2015 Aug 17;5:13068. doi: 10.1038/srep13068 (PMC4538375; doi:10.1038/srep13068)
Supplement: Supplementary Information [file srep13068-s1.pdf]

Supplementary Information:

The ubiquity and ancestry of insect *doublesex*

Dana C. Price[1,*] Andrea Egizi[1,2] and Dina M. Fonseca[1]

[1] – Rutgers University, Department of Entomology, New Brunswick, NJ, USA

[2] – Graduate Program in Ecology and Evolution, Rutgers University, New Brunswick, NJ, USA

* To whom correspondence should be addressed:

178 Jones Avenue

New Brunswick, NJ 08901 USA

d.price@rutgers.edu

Figure S1

Order	Species	GenBank accession	OD1 sequence	OD2 Sequence
Protura	*Acerentomon sp. AD2013*	GAXE01026579_4	TTPLCARCKNHGLKIKLKGHKRYCRYIRCI-CQECIATKARQIKMARQ	DASLVFYQQMEHIMQKFKLPIESLPAIYALFKDANGSVEE-TCEKLMSAYEDLHELSVRLEAL
Protura	*Acerentomon sp. AD2013*	GAXE01016537_1	<OD2 singleton>	AATLIFQQRLEHLLNKFKLPVESLPAIYAVLKDAKGCVEE-ASERLMAAYEELHEISVRLGLL
Zygentoma	*Atelura formicaria*	GAYJ01004010_4	PARLCAQCRNHGLRVPVKGHKRHCAYKQCT-CKECCLVKERQRVVALH	ARERMLYESLVMMRQAFPVGEEAMPLLLCILKHSR-SVQE-ASLKIYQGHYDLNAKGFVGLDG
Zygentoma	*Atelura formicaria*	FCD0KP1ACXX:7:2202:17946:4233:6[1]	<OD2 singleton>	-------KESLQTLLDMFRLPMETLPLIYVVLKDAR-SDVKEASNRIIEGGYWIMTQMAA
Zygentoma	*Tricholepidion gertschi*	GAS001256612_2	PSRLCAQCRNHGLRVPVKGHKRFCAYKLCH-CAECLLVKERQRVVALH	EREQMLYESLMVMRQAFPVGEEAMPLLLCILKHSR-SVQE-ASWKICQGTRDLSARGIIGIEN
Zygentoma	*Tricholepidion gertschi*	GAS001035827_5	<OD2 singleton>	EEGDVMMESLHALLEMFQLPLETLPLIYVVLKDARSDVKE-ASNRIIEAQSELRSIF------
Zygentoma	*Thermobia domestica*	GASN01031735_3	PSRLCAQCRNHGLRVPVKGHKRYCAYKLCT-CKECCLVKERQRVVALH	ARERMLYESLLVMRQAFPVGEEAMALLLCILKHSR-SVQE-ASWKIYQGTYDLTSRGFVGLDN
Zygentoma	*Thermobia domestica*	GASN01345779_5	<OD2 singleton>	ENVELLKDSLHALLDMFRLPLETLPLIYVVLKDARSDVKE-ASNRIMEGVYQCMNQGIIAPIG
Odonata	*Epiophlebia superstes*	GAVW01013444_6	TPPKCARCRNHRLKIPLKGHKRYCKFRYCK-CDKCRLTAERQRVMAMQ	ENMDFIKDAIVALLQMFRLPVDTLPLIYAVLKGTRCDVKE-ASNQIQDAQDDLRAMALRMMYP
Odonata	*Cordulegaster boltonii*	GAYO01003812_6	TPPKCARCRNHRLKIPLKGHKRYCKFRYCK-CDKCRLTAERQRVMAMQ	ENMDFIKDAIVALLQMFRLPVDTLPLIYAVLKGNRCDVKE-ASNQIQDAQDDLRAMALRMMYP
Ephemeroptera	*Ephemera danica*	GAUK01006643_5	SNRLCAQCRNHGLRVPVRGHKRYCTFRLCT-CRECRLVRERQRVVALH	TRERMLYESLMQLRQLYPVPEASLPLLLCILKSSR-SIDE-ASAKIVQGTHELSTRGFPGIEA
Ephemeroptera	*Isonychia bicolor*	GAXA01111429_5	SNRLCAQCRNHGLRVPVRGHKRYCTYRLCT-CRECRLVRERQRIVALH	TRERMLYESLLELRQSFPVPEAGLPLLLCILKSSR-SVHE-ASAKIVQGTHDLTTSGLLGLDG
Ephemeroptera	*Isonychia bicolor*	GAXA01058460_2	<OD2 singleton>	DNIDSLRDSIQTLLDTFRLPMETLPLVYVVLKVSRSDVQE-AFNRISEGGYHMTSSDVPVAAA
Ephemeroptera	*Baetis sp. AD-2013*	GATU01002143_4	SNRMCAQCQNHGLKIPVRGHKRFCKYRLCN-CQNCLLVKERQRIVALH	QRKNLMYSSLLELRSHHPVPDAALPLLLCILNISN-SVKE-ASEKIQKGLQELSLAAASSNEL
Zoraptera	*Zorotypus caudelli*	GAYA01001439_2	RTPKCGRCRNHGAINELKGHKDACRWKDCS-CSRCALLIARQKINQDL	DKTRVLQIINCLRSYHGLFSWESIPLVHIILEDCGMNYHA-ASSVIFNALRGQAVGRSSRGRT
Zoraptera	*Zorotypus caudelli*	GAYA01396307_3	AKPTCEKCKHHGVTVLLVGHKKRCPFTDNLKCAMCVKVDEKRLVMKEQ	KKEKLSKEVQALMKSFHYKSWESVPLLEIILEDTNFDINR-ASDLILKAYESQYRINVNAEKV
Zoraptera	*Zorotypus caudelli*	GAYA01394885_2	AKPTCEKCKHHGIIIFLEGHKNRCKYKDELDCEMCLAVDEKRRVMKDQ	KKEKLSKEVQTLMKSFDYKSWESVPLLEIILEDTNFDINK-ASDLILKAYENQYQIKVEEEKV
Zoraptera	*Zorotypus gurneyi*	GABA01003757_3	RIPMCGRCRNHGVINALKGHKDACRWKNCK-CSRCSLLIARQKINQDL	EKTRVLQIINCLRNFHGLSSWESIPLVHIILEDCGMNYHA-ASSVIFNALRRSCCGQEPPRKV
Zoraptera	*Zorotypus gurneyi*	GABA01001162_6	AKPTCEKCKHHNIIVLLDGHKKRCRYRNNLDCEMCIKVDEKRKVMKDQ	KKEKLRKEVHALMKSFGLKSWESVPLLEIILEDANFDVNK-ASDLILKAYESQYRMKVSAEKV
Dermaptera	*Apachyus charteceus*	GAUW01111996_3	-----------------------YRNCR-CNKCTLTAERQRIMAAQ	IKIQHIRESIHTIMTEFKLPSKSVVLIYTLLKLLRYSINN-VSHHINTSQTELEIIGNHEEQS
Dermaptera	*Forficula auricularia*	GAYQ01017360_5	KAPNCARCRNHYLKMGLKGHKRYCKFRYCE-CSKCRLTYERQRVMALQ	ANGDVLRDSISVIINKFNLTLEAVPMIYALLKSAHFSVHQ-VLENISNGKIPTSYCKINVVII
Orthoptera	*Tetrix subulata*	GASQ01124537_1	TPPNCARCRNHGLKIPLKGHKRYCKYRYHD-CDKCLLTAERQRVMALQ	ENFEVLRESIQALLEMFRYPVETLPLIYVILKYARSDIKE-ASNRILEAQEVVCAMGLRDAAR
Orthoptera	*Teleogryllus commodus*	GBHB01043004_5	RTPYCAKCRNHGLTVKLKGHKHKCRFRFHN-CPRCWVTTMRQHLTAIQ	-----LPQSIQMLLDAFRLPMDALPLVYMVLKDSRSDVKE-ASKRILEGERHSSRSAPPSTD-
Phasmatodea	*Aretaon asperrimus*	GAZQ01017692_2	MNRLCALCRNHGLKIPVKGHKRFCGYRLCL-CKECCLVKERQRVVAMH	VREQMLYESLLAMRRVFPVSDEAMPLLLCILKHSR-SLQE-ASWKIIQGTQSLISSGQKGLEL
Phasmatodea	*Aretaon asperrimus*	GAWC01030481_5	<OD2 singleton>	VSVEAMMDGVYTLLHMFHYHVEMLPLLVVLSDAHCDVSE-AYNRILQVLGNHLDSVLPVTSS
Phasmatodea	*Ramulus artemis*	GAWE01101273_2	TPPNCARCRNHRLKIGLKGHKRYCKYRYCT-CSKCRLTAERQRVMALQ	VSVEAMMDGVYTLLHMFHYHVEMLPLLVVLSDAHCDVTE-AYNRILQEAYPRRVGNDEKCVT
Phasmatodea	*Ramulus artemis*	GAWE01101272_2	TPPNCARCRNHRLKIGLKGHKRYCKYRYCT-CSKCRLTAERQRVMALQ	VSVEAMMDGVYTLLHMFHYHVEMLPLLVVLSDAHCDVTE-AYNRILQGECAAA---------
Phasmatodea	*Medauroidea extradentata*	GAWD01075183_3	MNRLCALCRNHGLKIPVKGHKRFCGYRLCL-CKECCLVKERQRVVAMH	VRDQMLYESLLAMRRVFPVGDEAVPLLLCILKHSR-SLQE-ASWKIIQGTQSLISSGHKGYES
Phasmatodea	*Extatosoma tiaratum*	GAWG01081702_1	INRLCALCRNHGLKIPVKGHKRFCGYRLCL-CKECCLVKERQRVVAMH	VREQMLYESLLAMRRVFPVGDEAVPLLLCILKHSR-SLQE-ASWKIIQGTQSLISSGHKC*--
Phasmatodea	*Sipyloidea sipylus*	GAWF01025783_1	INRLCALCRNHGLKIPVKGHKRFCGYRLCL-CKECCLVKERQRVVAMH	VREQMLYESLLAMRRVFPVGDEAVPLLLCILKHSR-SLQE-ASWKIIQGTQTLISSGQKGTEA
Phasmatodea	*Peruphasma schulteyi*	GAWJ01240650_4	MNRLCALCRNHGLKMPVKGHKRFCGYRLCL-CKECCLVKERQRVVAMH	VREQMLYESLLAMRRVFPVGDEAVPLLLCILKHSRSLQEA-SWKIIQGTQSLISNGHKGLEAF
Phasmatodea	*Timema cristinae*	GAVX01147206_3	LNRLCALCRNHGLKKPVKGHKRFCAYKLCV-CRECCLVKERQRVVALH	VREQMLYESLLAMRRVFPVGEEAVPLLLCILKQSRSLHEA-SWKIIQGTQTLISRGHTGLDSY
Mantodea	*Metallyticus splendidus*	GATB01322676_2	TPPNCARCRNHRLKIGLKGHKRYCMFRHCT-CTKCRLTAERQRVMAMQ	ESMEIPWDAALTLLEKFGLPAVSLPLIYVVLQYSRSDVSE-ASRHIVSGKYLLR---------
Mantodea	*Mantis religiosa*	GASW01222710_6	TPPNCARCRNHRLKIGLKGHKRYCKYRYCN-CDKCCLTAERQRVMALQ	ESITVSRESILELLERFRLPWETLPLIYVVLDDARCDVSE-ASKRILKGYYVNTSHKVINTFK
Mantodea	*Mantis religiosa*	GASW01220805_1	TPPNCARCRNHRLKIGLKGHKRYCMYRTCM-CTKCRLTAERQRVMAMQ	DTMDIPWEAVLALLQKFEFPAVSLPLIYVVLQYARSDVSE-ASRHILTGKYLIAMQFSP*---
Hemiptera	*Cercopis vulnerata*	GAUN01115682_1	TPPNCARCRNHGLINILKGHKRYCPFVDCT-CEKCSLTLERQKVMAKQ	ENSKEPWDSILLLMHMLGLPVNTTPLLIILKEVASDAYE-VCNRFFAAQEELRSTCPIKEEI
Hemiptera	*Okanagana villosa*	GAWQ01014169_1	TPPNCARCRNHSLILPLKGHKRYCKYFDCD-CDKCELTAQRQKVMAKQ	RDSKELWESILHLLHWFSLPVNTTPLLHIILNEVATDEFD-AYNRILDAQEELRITCPIKEEI
Hemiptera	*Okanagana villosa*	GAWQ01014168_1	TPPNCARCRNHSLILPLKGHKRYCKYFDCD-CDKCELTAQRQKVMAKQ	RDSKELWESILHLLHWFSLPVNTTPLLHIILNEVATDEFD-AYNRILDGKHSCTVSIIQNTKH
Hemiptera	*Notostira elongata*	GASV01006917_3	KQRFCQFCRNHTKMIRTKGHKKECDYRFCK-CIKCRNTRERHKVMAKQ	TSLRIMWERILTVLIEKGYSLDTYPLLYIILKEVTSDVEE-VYQKIAQWTESSQFILVI*---
Hemiptera	*Xenophysella greensladeae*	GAYI01023103_2	-APNCARCANHCIREPLKGHKRFCGFRDCD-CQKCQLTAERQRVMAQQ	GGEEVHWESITALLEMFQYRLGPCPLLHIILNKITPNIKE-AYSLILAGRPQLSFIN*-----
Psocodea (Phthiraptera)	*Menopon gallinae*	GAWR01005396_5	TPPNCARCRNHDIKIALKNHKRYCRYKHCK-CTKCILTLQRQTVMAAQ	KSLENHRNCVMKLVKDFGLGIDAYPLAYVVFKIAGGDYEV-ATKMILEAQEEITKMSLLSASM
Psocodea (Psocoptera)	*Liposcelis bostrychophila*	GAYV01012131_4	TPPNCARCRNHDIKVGLKNHKRYCKFKLCK-CAKCILTLQRQKVMAAQ	KNLKNYRYYVMKLVTDFGLSIDSYPLVLVIFKILGGDYDL-AAKMITEGCDEVYPCLG-----
Hymenoptera	*Cotesia vestalis*	GAUP01001251_3	TPPNCARCRNHRLKIALKGHKRYCKYRNCN-CEKCILTKDRQRVMALQ	ENVEVLLECSTKLLERFWYSWDMLPLMYVILKDARADIEE-ATRRIAEANTEIRAVAFWKARR
Hymenoptera	*Chrysis viridula*	GATY01010708_4	ALRSCARCSNHRLKIALKGHKHYCKYRLCN-CKKCRLTAERQKVMAKQ	DSVEVLLECSSKLLELFQYPWDMVPLMYVILKDAGANLEE-AVRRIVEANNDIRALHFWKEVR
Hymenoptera	*Orussus abietinus*	GAUJ01013146_6	GTARCSRCLNHEVNMPLKGHKRYCFYKYCI-CTKCLSTAERQVIMAAE	ESMTDLLQLTLELLNKFEYTWDTIPLMYLILKSVMGDREK-ASRLIREVNDEIRKIYNYHCNR
Hymenoptera	*Tenthredo koehleri*	GAWW01003430_6	TPPNCARCRNHRLKIALKGHKRYCKYRSCN-CEKCRLTAERQRVMALQ	ENVEVLLEYSTKLLERFRYPWEMLPLMLVILKDAQADLEE-ASRRIAEENIKPAKLAYNPMII
Hymenoptera	*Microplitis demolitor*	JO916215_4	TPPNCARCRNHRLKIALKGHKRYCKYRNCN-CEKCILTKDRQRVMALQ	ENVEVLLECSTKLLERFWYSWDMLPLMYVILKDARADIEE-ATRRIAEGEENLIFKLRIHISL
Hymenoptera	*Sphaeropthalma orestes*	GAXP01022687_1	SRPSCARCRNHDLHIRLKGHKRYCRYLACT-CPKCLLTADRQRVMAKQ	GDIEVLLDYSTKLLELFHYPWETLPLIYVIVKGLGPNVEE-AVRRIVEASNEIRMINFWKAFR
Hymenoptera	*Sphaeropthalma orestes*	GAXP01017427_3	RKPCCARCRNHRVKVALKGHKRICPYKSCK-CPKCEITADRQKITAMQ	DNVEIMLQYSMKLLELFQYSLKMLPLMYLIVKDSGANLEE-AVRRIVEANNEIRTMHFWKAMR
Hymenoptera	*Osmia cornuta*	GAGH01066955_2	KPPNCARCRNHQLKIAVKGHKRYCRYRYCK-CDSCKLTADRQRVMARQ	DTVEIMLEYSTKLLELFQYPWEMLPLMYVILKDAGGNLEE-AMRRIVE--------------
Raphidioptera	*Xanthostigma xanthostigma*	GAUI01017467_1	TPPNCARCRNHRVKIPLKGHKRYCKYRTCN-CQKCRLTAERQRVMAMQ	HNVEVVLNYCHRLLDTFSYPWEMMPLMYVILKYASGDLTE-AVRSIDEGKRVVNEYSRIHNLN
Raphidioptera	*Inocellia crassicornis*	GAZH01013937_4	TPPNCARCRNHRVKIPLKGHKRYCKYRTCS-CQKCRLTAERQRVMAMQ	HNVEVVLNYCHRLLDTFSYPWEMMPLMYVILKYASGDLEE-AVRSIDEGKSVMSSIFYKMEMT
Megaloptera	*Corydalus cornutus*	GATG01001294_4	TRPNCARCRNHRVKVPLKGHKRYCKYRTCS-CQKCRLTAERQRVMAMQ	NNIELILDCCHRLLDTFLYPWELMPLMYVILKYAGGNLDE-AIRSIREGKRVVNEYSRTHNLN
Neuroptera	*Conwentzia psociformis*	GAYH01007785_3	APPNCARCNNHKLKIPLKGHKRYCRYKRCC-CSKCLLTLSRQQDMAKQ	VPSEAIIDKCHALLDKFKYPWEIMHLLITILRMCNYDYKL-AETQIQEGKEIVNEYSRTHNLN
Neuroptera	*Osmylus fulvicephalus*	GAYC01086997_6	TPPNCARCRNHRMKVPLRGHKRYCNFRNCT-CQKCRLTADRQRVMAMQ	KQEEVVLEYCFRLLDRLKYPWEVMPLLYAILNVCDWNMDE---------------------
Strepsiptera	*Stylops melittae*	GAZM01003841_2	TPPNCARCRNHLLKIPLKGHKRYCRYRTCA-CNKCCLTSERQRVMAMQ	INTEQIIDYSQKLMEIYKYPYEMMPLMYAILKSVNVDFIE-AQTRIDEGHQVVSDFLRKHNLN
Coleoptera	*Meloe violaceus*	GATA01048201_6	TPPNCARCRNHRMKVALKGHKRYCKFRTCK-CEKCRLTSERQRVMAMQ	AQSTDLLEDCQKLLERFKYPWEMMPLMYAILKDARADLEE-ASRRIDEGKRVVNEYSRIHNLN
Coleoptera	*Meloe violaceus*	GATA01002860_6	<OD2 singleton>	RGAENLLEFCQRLKDKFQLSWKMISLVDVILKYAKEDQEE-AWRQIDEAFLEIRALAAVEAAR
Coleoptera	*Gyrinus marinus*	GAUY01088671_2	TPPNCARCRNHRLKIALKGHKRYCKYRACK-CEKCRLTSERQRVMAMQ	GQSTELLEDCQKLLDRFKYPWEMMPLMYVILKDAKADLEE-ASRRIDEGKRAVYEYSRIHNLN
Coleoptera	*Gyrinus marinus*	GAUY01088671_3	<OD2 singleton>	RSLCILHEICQKLHERYQWSWKMFPLVYLILKTHNEDYGD-ITRRIDEAYQEIRAIVAVETAK
Coleoptera	*Meligethes aeneus*	GAPE01021632_6	TPPNCARCRNHRKKIALKGHKRYCMYRTCK-CEKCLLTTERQRVMAMQ	HQSTDLLEDCQKLLERFKYPWEMMPLMYAILKDARADLEE-ASRRIDEGRGFEILLDLCHRLR
Coleoptera	*Meligethes aeneus*	GAPE01021632_6	<OD2 singleton>	RGFEILLDLCHRLREKFNLSWRMIPLVDVILKYAKENQDE-AWRQIEEAFLYIKERTLAALEV
Coleoptera	*Aleochara curtula*	GATW01000814_5	TPPNCARCRNHRLKVGLKGHKRYCTFRHCK-CEKCKLTAERQRVMAMQ	VHSTDLMEDCLKLLERFRYPWEMLPLMYTILKDANGDLEE-AARRIDDGFIVLKTLLQTVVGY
Coleoptera	*Pogonus chalceus*	JU430525_5	TPPNCARCRNHRLKIALKGHKRYCKYRSCK-CEKCKLTSERQRVMALQ	GQSTELLEDCQKLLDRFKYPWEMMPLMYVILKDAQADIEE-ASRRIDEDSNIKLLADLCSRLS
Coleoptera	*Dendroctonus frontalis*	GAFI01011207_3	TPPNCARCRNHRKKITLKGHKRYCMYRNCK-CQKCQLTSERQRVMAMQ	DRSMDLLEDCHKLLEKFQYPYEMMPLMYAILKDARADLDE-ASRRIDEGKQVVNEYSRTHNLN
Trichoptera	*Annulipalpia sp. AD-2013*	GATX01084595_6	TPPNCARCRNHRLKIALKGHKRYCKYRYCN-CDKCRLTAERQRVMALQ	PLFENMLDNCQKLIEKFQYSWEMMPLIYVILKYAKGNTEE-ASKSIEEGKWVITEYARQNNLN
Lepidoptera	*Nemophora degeerella*	GATC01014479_1	APPNCARCRNHRVKVELKGHKRYCKYRLCN-CDKCRLTADRQRVMALQ	HSLEALVENCHKLLEKFHYSWEMMPLVLVILNYAKSDIEE-ASRRIDEGKKIVNDYAWKHNLN
Lepidoptera	*Parides eurimedes*	GAXH01085310_6	APPNCARCRNHRLKVELKGHKRYCKYRYCN-CEKCRLTADRQRVMAMQ	VSLETLVENCHRLLEKFHYSWEMMPLVLVILNYAGSDLDE-ASRKIDEGKLIVNEYARKHNLN
Lepidoptera	*Spodoptera litura*	GBBY01009273_1	APPNCARCRNHRLKIELKGHKRYCKYRNCT-CEKCILTADRQRVMAQQ	VSLENLVDNCNKLLEKFHYSWEMMPLVLVILNYAGSDVDE-ASRKIDEGKMIINEYARKNNLN
Lepidoptera	*Trichoplusia ni*	GBKU01001268_4	APPNCARCRNHRLKVELKGHKRYCRYRHCT-CEKCRLTADRQRVMALQ	VSLDALVENCHKLLEKFHYSWEMMPLVLVILNYAGSDLEE-ASRKIDEGKLMIDEYARKHNLN
Lepidoptera	*Helicoverpa armigera*	GBDM01028004_1	APPNCARCRNHRLKIELKGHKRYCKYRNCT-CEKCRLTADRQRVMALQ	VSFETLVENCQKLLDKFHYSWEMMPLVLVILNYAGSDLDE-ATRKIDEGKMIINEYARKHNLN
Siphonaptera	*Ceratophyllus gallinae*	GAWK01054083_5	TPPNCARCRNHRLKIALKGHKRYCRFLYCK-CEKCKLTADRQRVMAKQ	EEVKTLLEESHQILEQLKYPFEMLPFVFAILRKSDG-ISD-AIKNIHEGQLVVSEYSRMNNLN
Siphonaptera	*Ctenocephalides felis*	GAYP01008255_1	TPPNCARCRNHRLKIPLKGHKRYCRYLYCK-CEKCRLTADRQRDMARQ	EKVKALLEESQQIVEQLKYPFEMLPFVYAILGLSGG-ISE-AIKNIHNGQLVVSEYSRMNNLN
Siphonaptera	*Oropsylla silantiewi*	GAWY01012876_1	TPPNCARCRNHRLKIALKGHKRYCRFLYCK-CEKCRLTADRQRVMAKQ	EEVKILLEESHQILEQLKYPFEMLPFVFAILRKSNG-ISD-AIKNIHEAVQAVQLSFFRGWFD
Mecoptera	*Boreus hyemalis*	GAYK01000638_3	TPPNCARCRNHRLKIALKGHKRYCKFRNCN-CYKCILTAERQRIMAVQ	SDARDYVQMCYRLLDHFNYPFEMMPLIYANLKYAGN-IDE-ASRRIEEGQWVVSEYSRLNNLN
Mecoptera	*Boreus hyemalis*	GAYK01015512_3	<OD2 singleton>	YSDKVVFDLSQKLLEKFNYPFEMMPLIYANLKYAGS-IDE---------------------
Mecoptera	*Boreus hyemalis*	GAYK01017535_4	<OD2 singleton>	YFDKVVFDLSFKLLKKFNCPIEMMPLIYANLRYADN-IDE-ASRRIEEDILRITYGRYHLVNL
Diptera	*Bombylius major*	GATI01007470_4	TPPNCARCRNHGLKIALKGHKRYCKYRYCN-CEKCRLTAERQRVMALQ	QNAELLLEYCQKLLEKFRYPWEMMPLMYVILKDARADIEE-ATRRIDEGQCVVNQYSRIHNLN
Diptera	*Bombylius major*	GATI01007471_5	<OD2 singleton>	QKAERLLEYCQKLLEKFRYSWKMMPVMYAILKDARADVEE-ASRRIDEAIRVVRPLTLHLRYP
Diptera	*Triarthria setipennis*	GAVA01013308_3	TKPNCARCHNHGFKIQLKGHKRYCKFRNCN-CDKCRLTADRQRVMALQ	MSIDLILDYCQKLIEKFGYPWEMMPLMYVILKDAGVDIDE-ASKRIEEGIQVLKDSKMFDGID
Diptera	*Trichocera saltator*	GAXZ01015564_6	TPPNCARCRNHSLKIALKGHKRYCRFRHCS-CEKCRLTAERQRVMALQ	QNTELLLEYCQKLLDKFNYPWEMMPLMYAILKNENN-VEE-AERRIDEGRYVFKLAMRNHVMS
Diptera	*Liparia lucens*	GAZD01014428_3	TPPNCARCRNHGLKITLKGHKRYCKYRYCR-CEKCRLTADRQRVMALQ	TEDPQFLYNCEKLLEKLRYPWEMMPLIFAILKSARNDIEE-ALKLIEEGQHVVNEYYRQHNLN
Diptera	*Drosophila miranda*	GALP01005259_3	TPPNCARCRNHGLKITLKGHKRYCKYRYCT-CEKCRLTADRQRVMALQ	LGQDVFLDYCQKLLEKFRYPWELMPLMYVILKDADANIEE-ASRRIEEGQYVVNEYSRQHNLN
Diptera	*Sitodiplosis mosellana*	GAKJ01011849_2	TPPNCARCRNHGDKVELKGHKRYCKYRYCN-CDKCNLTAERQRVMALQ	DNADMLLEKSQKMIERFNYPWEMMPLLYVILKYANGDLEE-ATNQLSEGQDFIGKYSRMHNLN
Diptera	*Teleopsis dalmanni*	GBBP01063334_1	TPPNCARCRNHGLKIILKGHKRYCKFRFCT-CEKCRLTVERQINMAQK	PPRDILIGNSEKLLQKFGYPYELMPLMYVIVRYAKADMEE-ASRSIDEGIRDTKDYKNLIDNL
Diptera	*Bactrocera dorsalis*	GAKP01007048_1	TPPNCARCRNHGLKITLKGHKRYCKFRFCT-CEKCRLTADRQRVMALQ	LAQDVFLEHCQKLLEKFRYPWEMMPLMYVILKDAGADIEE-ASRRIEEGQHVVNEYSRQHNLN
Diptera	*Ceratitis capitata*	GAMC01004171_3	TPPNCARCRNHGLKITLKGHKRYCKFRYCT-CEKCRLTADRQRVMALQ	LAQDVFLEHCQKLLEKFRYPWEMMPLMYVILKDAGADIEE-ASRRIEEAKRIVNQTISLHWMD
Diptera	*Anopheles quadrimaculatus*	GBTE01014262_1	TPPNCARCRNHGLKIGLKGHKRYCKYRNCN-CEKCCLTAERQRVMALQ	LPDDELVKRAQWLLEKLSYPWEMMPLMYVILKSADGDVQK-AHQRIDEGKRTIKAYELVKSSL
Diptera	*Aedes albopictus*	GAPW01003320_1	TPPNCARCRNHGLKIGLKGHKRYCRYRHCS-CEKCCLTAERQRVMALQ	RTDDELVRQSQQLLEKLHYPWEMMPLMYVILKGANGDVAK-AHQRIDEGQAVVNEYSRLHNLN
Diptera	*Psorophora albipes*	GALA01000925_1	TPPNCARCRNHGLKIGLKGHKRYCKYRNCN-CEKCCLTAERQRVMALQ	RTDDELVKRSQYLLEKLHYPWEMMPLMYVILKGADGDVQK-AHQRIDEGMGVWRCWDFRWVVN
Diptera	*Anopheles funestus*	EZ974425_2	TPPNCARCRNHGLKIGLKGHKRYCKYRTCH-CEKCCLTAERQRVMALQ	LPDDELVKRAQWLLEKLGYPWEMMPLMYVILKSADGDVQT-AHQRIDEGQAVVNEYSRLHNLN
Diptera	*Corethrella appendiculata*	GANO01004847_1	TAPNCARCRNHALKIPLRGHKRFCKYRYCG-CEKCRMTAERQRDMAHK	MQNDILLEKSHQLLEKFNYPWELLHLMYTILKYSRANIEE-AARRIEEGQYVVNEYSKLHNLN
Diptera	*Culicoides sonorensis*	GAWM01002290_1	TPPNCARCRNHGLKITLKGHKRYCRYRYCN-CEKCRLTADRQKIMALQ	RKTDRLIDNCDTLCSRYGYPWEMMPLVYTILALVNGDIEE-ACNRIDEGKMAILDYSNQNNVS
Diptera	*Belgica antarctica*	GAAK01007185_1	TPPNCARCRNHGLKIGLKGHKRYCKHRYCN-CDKCRLTAERQRVMALQ	NNNDNFWDMADQMARDNNYPDEVTPLLHALIKSRKGKFHE-AQKLLDEGKMVVTEYSRRLNIN
Diptera	*Belgica antarctica*	GAAK01003177_1	KGSNCVRCRNHNLTVPLRGHKQYCPFAACT-CDKCCFTAEQQRQMRLL	KDRKALFDCSSELLKKFRYPWEMMPLMYALSKYANGNIGE-VLQRIDEGQFAVNEQLRQQTLN

Figure S2

Order	Species	[Accession]_[frame]/[range]	Sequence	HMM e-val
Protura	*Acerentomon sp. AD-2013*	GAXE01105806.1_2/58-86	RLPKCARCINHGLKIPLKGHKRYCTNKYCDCP---------------	8.5e-13
Collembola	*Sminthurus viridis*	FCD0GUKACXX:7:1301:17137:108047:3[1]	TPPNCARCRNHRLKIPLRGHKRYCRFRTCKCQKCILTAERQRVMAMQ	2.0e-14*
Archaeognatha	*Lepismachilis y-signata*	Contig_383881[2]	APPNCARCRNHRLKVELKGHKRYCKYRYCNCEKCRLTADRQRVMALQ	7.2e-28*
Zygentoma	*Thermobia domestica*	GASN01365969.1_4/81-127	TPPKCARCRNHRLKIPLKGHKRYCKYRFCNCDKCLLTAERQRVMALQ	1.3e-29
Odonata	*Calopteryx splendens*	GAYM01080693.1_4/36-82	TPPKCARCRNHLYKIPLKGHKRYCKFRYCKCDKCCLTAERQRVMAKQ	1.4e-29
Ephemeroptera	*Isonychia bicolor*	GAXA01091320.1_6/79-119	TPPKCARCRNHRLKIPLRGHKRYCNYRYCNCEKCKLTADRR------	1.9e-24
Ephemeroptera	*Baetis sp. AD-2013*	GATU01010243.1_2/88-133	SNRMCAQCQNHGLKIPVRGHKRFCKFRLCNCQNCLLVKERQRIVALH	1.2e-20
Ephemeroptera	*Eurylophella sp. AD-2013*	GAZG01080309.1_5/8-52	ASRLCAFCRNHSLKIPVKGHKRFCRNRTCNCAECKLVRERQRVVALH	1.9e-20
Dermaptera	*Apachyus charteceus*	GAUW01001274.1_3/14-59	KTPNCARCRNHYVKIKLKGHKRYCKYRNCRCNKCTLTAERQRIMAAQ	4.8e-28
Dermaptera	*Forficula auricularia*	GAYQ01017359.1_5/107-152	KAPNCARCRNHYLKMGLKGHKRYCKFRYCECSKCRLTYERQRVMALQ	2.3e-27
Plecoptera	*Leuctra sp. AD-2013*	GAUF01079838.1_5/141-187	TPPKCARCRNHDVKITLKGHKRFCAFRFCNCEKCCLTVERQRVMAAQ	3.0e-27
Plecoptera	*Cosmioperla kuna*	GAYL01095698.1_6/28-74	TPPNCARCRNHLLKIALKGHKRFCRYRYCECDKCRLTAERQRVMAAQ	3.4e-29
Grylloblattodea	*Grylloblatta bifratrilecta*	GAWP01142412.1_3/58-104	TPPNCARCRNHRLKIGLKGHKRYCKYRASPCDKCCLTAERQRVMAMQ	5.4e-29
Grylloblattodea	*Galloisiana yuasai*	GAWN01171825.1_4/47-93	TPPNCARCRNHRLKIGLKGHKRYCKYRHCTCDKCCLTAERQRVMAMQ	2.0e-30
Embioptera	*Haploembia palaui*	GAZA01236824.1_5/84-130	TPPNCARCRNHNFKIGLKGHKRYCRFRSCTCSKCCLTAERQRVMALQ	3.3e-28
Mantodea	*Empusa pennata*	GAWT01309511.1_4/111-157	TPPNCARCRNHRLKIGLKGHKRYCKYRYCNCDKCCLTAERQRVMALQ	2.8e-30
Blattodea	*Periplaneta americana*	GAWS01015027.1_4/1-44	---NCARCRNHRLKIGLKGHKRYCKYRYCNCDKCCLTAERQRVMALQ	1.2e-28
Blattodea	*Blatella germanica* genome	JPZV01136765.1_2/621-668	TPPNCARCRNHRLKIGLKGHKRYCTFRSCVCEKCVLTAERQRVMALQ	1.7e-15*
Isoptera	*Prorhinotermes simplex*	GASE01075660.1_4/15-55	KIKYCSKCSAHTTKVQIKNHKNKCPFKDCLCKDCIPIIVRRDSTALA	7.6e-08
Hemiptera	*Xenophysella greensladeae*	GAYI01136691.1_3/24-70	TAPNCARCRNHGQKIPLKGHKRYCCFRDCDCEMCCLTAKRQKVMALQ	1.7e-27
Hemiptera	*Planococcus citri*	GAXF01116661.1_1/146-191	TAPNCARCKNHGLKIPLKGHKRYCRFWNCVCEKCVQTSTRQKVMARE	1.2e-22
Hemiptera	*Planococcis citri*	GAXF01103858.1_1/43-88	TPPNCARCKNHGLKNVLSGHKRYCLYLECKCEKCLATVGRQRSMAKN	4.9e-20
Hemiptera	*Acanthosoma haemorrhoidale*	GAUV01009568.1_3/70-108	KVKYCAKCKNHGRKEEMKGHKRECERRHCSCDKCQATEKKRAKTRIQ	7.1e-12
Psocodea (Psocoptera)	*Ectopsocus briggsi*	GAPT01011620.1_2/92-137	KIPYCGRCRNHGIKEVLKGHKKKCIYRDCTCGSCMLSVNRREIAKKQ	8.3e-20
Psocodea (Psocoptera)	*Ectopsocus briggsi*	GAPT01002163.1_6/155-201	TPPICNRCKVHGVKQSLKGHKRYCRFLTCDCSLCKLVVEKRVVMADQ	4.3e-15
Neuroptera	*Pseudomallada prasinus*	GAVV01019538.1_2/82-128	TPPNCARCRNHRLKIPLRGHKRYCRFRNCICHKCKLTAERQRVMAMQ	2.2e-28
Neuroptera	*Conwentzia psociformis*	GAYH01007786.1_3/137-182	APPNCARCNNHKLKIPLKGHKRYCRYKRCCCSKCLLTLSRQQDMAKQ	3.7e-22
Neuroptera	*Euroleon nostras*	GAXW01087396.1_4/25-71	TPPNCARCRNHRLKIPLRGHKRYCRFRTCTCEKCRLTAERQRVMAMQ	7.1e-29
Coleoptera	*Lepicerus sp. AD-2013*	GAZB01138597.1_2/45-91	TLPNCARCRNHSIKEPLKGHKRYCKFKKCQCEKCKLTAERQRVMAKQ	6.4e-28
Trichoptera	*Rhyacophila fasciata*	GAXX01046516.1_4/10-56	TPPNCARCRNHGLKIGLKGHKRYCKYRYCTCEKCRLTAERQRVMALQ	4.8e-31
Trichoptera	*Platycentropus radiatus*	GASS01092876.1_3/184-204	TPPNCARCRNHRLKIALKGHK--------------------------	4.0e-08
Trichoptera	*Hydroptila sp. AD-2013*	GAVM01073298.1_1/112-158	TPPNCARCRNHRLKIALKGHKRYCKFRHCNCDRCRLTAERQRVMALQ	3.6e-29
Lepidoptera	*Yponomeuta evonymellus*	GASG01093141.1_4/23-68	APPNCARCRNHRLKVELKGHKRYCKYRYCNCEKCRLTADRQRVMALQ	3.2e-30
Lepidoptera	*Polyommatus icarus*	GAST01056365.1_4/55-86	APPNCARCRNHRLKIQLKGHKRYCKYRYCTCEK--------------	1.1e-19
Lepidoptera	*Dyseriocrania subpurpurella*	GASY01015364.1_1/256-302	TPPNCARCRNHQVKVALKGHKRYCRYRYCNCDKCRLTADRQRVMAMQ	2.5e-29
Lepidoptera	*Trodia sylvina*	GAVB01150705.1_2/60-106	TPPNCARCRNHRVKEKLKGHKRYCKYRYCNCDKCKLTAERQRVMALQ	1.9e-30
Siphonaptera	*Ctenocephalides felis*	GAYP01006643.1_1/27-73	TPPNCARCRNHRLKIALKGHKRYCRYLYCKCEKCRLTADRQRVMAKQ	6.6e-29
Mecoptera	*Panorpa vulgaris*	GAUH01062093.1_3/57-103	TPPNCARCRNHRLKIALKGHKRYCTFKFCNCEKCRLTAERQRVMALQ	1.5e-28
Diptera	*Bibio marci*	GATJ01056020.1_6/139-184	TQPNCARCRNHCYKVLLKGHKHYCRFRYCKCEKCVVTAKRQDIMANE	2.7e-25

Figure S3

Order	Species	[Accession]_[frame]/[range]	HMM aligned sequence	HMM e-val
Archaeognatha	*Meinertellus cundinamarcensis*	GAUG01133643.1_3/44-81	-TPKCARCRNHGVISCLKGHKRLCRWRECRCPNCLLVVE--------	5.6e-15
Blattodea	*Periplaneta americana*	GAWS01189376.1_5/2-47	-TPKCARCRNHGVISCLKGHKRLCRWRECQCPNCQLVVERQRVMAAQ	7e-21
Blattodea	*Cryptocercus wright*	GAZN01162439.1_6/11-56	-RPKCARCRNHGMISWLKGHKRQCRFKSCVCAKCNLIAERQRVMAAQ	1.2e-20
Calanoida	*Calanus finmarchicus*	GAXK01154012.1_1/25-70	-RPKCARCRNHGLISWLKGHKRSCQYRDCDCAKCNLIQERQRVMAAQ	3.1e-21
Calanoida	*Calanus finmarchicus*	GAXK01154011.1_1/25-70	-RPKCARCRNHGLISWLKGHKRSCQYRDCDCAKCNLIQERQRVMAAQ	3.2e-21
Coleoptera	*Aleochara curtula*	GATW01017006.1_5/27-72	-VPKCARCRNHGMISTLRGHKKQCIYKNCSCAKCGLIKERQRIMAAQ	4.6e-20
Coleoptera	*Ips typographus*	GACR01003199.1_2/53-98	-VPKCARCRNHGMISTLRGHKKQCIYKGCNCAKCGLIKERQRIMAAQ	7.4e-20
Collembola	*Tetrodontophora bielanensis*	GAXI01024987.1_5/8-53	-TPKCARCRNHGVISCLRGHKKMCRWKECTCEMCELVAERQRIMAAQ	8.8e-21
Collembola	*Pogonognathellus sp. AD-2013*	GATD01092185.1_3/9-54	-TPKCARCRNHGVISCLRGHKKMCRWKECSCEMCELVAERQRIMAAQ	2.1e-19
Collembola	*Anurida maritime*	GAUE01052744.1_3/128-173	-APKCARCRNHGVISCLRGHKKMCRWKECDCELCELVAERQRIMAAQ	1.2e-18
Diptera	*Culicoides sonorensis*	GAWM01017604.1_1/28-73	-TPKCARCRNHGVISCLKGHKRYCRWKECTCPHCLLVVERQKIMAQQ	5e-20
Diptera	*Trichocera saltator*	GAXZ01130618.1_6/9-54	-TPKCARCRNHGVISCLKGHKKLCRWKECCCPNCQLVVERQRVMAAQ	2.2e-19
Ephemeroptera	*Isonychia bicolor*	GAXA01106317.1_1/122-167	-TPKCARCRNHGVISGLKGHKRLCRWRECRCQSCLLVVERQRVMAAQ	5e-21
Ephemeroptera	*Eurylophella sp. AD-2013*	GAZG01012200.1_6/32-77	-SPKCARCRNHGVISSLKGHKRLCRWRECRCPSCLLVVERQRVMAAQ	4.7e-20
Grylloblattodea	*Galloisiana yuasai*	GAWN01063629.1_6/2-47	-RPKCARCRNHGMISWLKGHKRQCRFKNCVCAKCNLIAERQRVMAAQ	4.2e-21
Hemiptera	*Graminella nigrifrons*	GAQX01025711.1_6/87-133	TPPNCARCRNHDIIVQLKGHKRYCGYISCRCEKCLLTAERQKVMAKQ	3.6e-24
Hemiptera	*Cercopis vulnerata*	GAUN01102337.1_4/98-144	TSPNCARCRNHGIINILKGHKRYCPYVDCSCEKCSLTMERQKVMAKQ	9.7e-23
Hemiptera	*Macrosiphum euphorbiae*	GAOM01011235.1_1/7-52	-RPKCARCRNHGVISWLKGHKKRCSYKECVCPKCNLIAERQRVMAAQ	2.4e-19
Hemiptera	*Cercopis vulnerata*	GAUN01003127.1_4/8-53	-TPKCARCRNHGVISCLKGHKRLCRWKDCHCPNCQLVVERQRGMAAQ	2.8e-18
Hemiptera	*Nilaparvata lugens*	GAYF01062266.1_2/4-49	-HPKCARCRNHGMISWLKGHKKQCMFKSCSCA-CSLIAERQRVMAAQ	1e-15
Hemiptera	*Acanthocasuarina muellerianae*	GAYY01137565.1_1/301-346	TLPNCARCRYHKVIVGLKDHKHYCPNKTCKCEGCLNIEERQRVMAA-	4.4e-16
Hemiptera	*Cercopis vulnerata*	GAUN01092972.1_3/100-120	-PPNCARCRNHDIINILKGHKR-------------------------	1.2e-05
Hymenoptera	*Ganaspis sp. G1*	GAIW01023601.1_3/89-135	TKPNCARCRNHKIIIKLKQHKNFCKYRYCMCEKCILLKERQRIMAEQ	1.9e-22
Hymenoptera	*Ganaspis sp. G1*	GAIW01012036.1_5/215-260	-STNCGYCRNHNIIIGLKGHKIFCKYRYCTCEKCILLKERQRIMAEQ	1.5e-20
Hymenoptera	*Leptopilina clavipes*	GAXY01053312.1_1/30-75	-KPKCARCRNHGLISWLRGHKRECRYRDCFCVKCSLIAERQRVMAAQ	1.5e-20
Hymenoptera	*Stigmatomma oregonense*	GAXR01000833.1_2/51-96	-RPKCARCRNHGLISWLRGHKRECRYRECLCPKCALIAERQRVMAAQ	2.2e-20
Hymenoptera	*Ganaspis sp. G1*	GAIW01012035.1_5/481-526	-STNCGYCRNHNIIIGLKGHKIFCKYRYCTCEKCILLKERQRIMAEQ	4.7e-20
Hymenoptera	*Cotesia vestalis*	GAUP01064990.1_1/26-71	-SPKCARCRNHGIISSLKGHKRSCRWKDCNCPCCLLVVERQRVMAAQ	1.2e-19
Hymenoptera	*Cotesia vestalis*	GAUP01004774.1_1/35-80	-RPKCARCRNHGLISWLRGHKRECRYKECLCPKCSLIAERQRVMAAQ	2e-19
Hymenoptera	*Cotesia vestalis*	GAKG01004932.1_5/23-68	-SPKCARCRNHGIISSLKGHKRSCRWKDCNCPCCLLVVERQRVMAAQ	3.1e-19
Hymenoptera	*Osmia cornuta*	GAGH01047348.1_5/369-414	-RPKCARCRNHGLISWLRGHKRECRYRECLCPKCSLIAERQRVMAAQ	2.7e-19
Hymenoptera	*Mischocyttarus flavitarsis*	GAXM01016837.1_6/60-105	-SPKCARCRNHGVISGLKGHKRSCAWKDCRCPCCLLVVERQRVMAAQ	7.6e-19
Hymenoptera	*Sphaeropthalma orestes*	GAXP01040437.1_2/70-113	-RPKCARCRNHGLISWLRGHKKECRYRECLCVKCSLIAERQKVMA--	1e-19
Hymenoptera	*Stigmatomma oregonense*	GAXR01010442.1_4/19-64	-SPKCARCRNHGVISGLKGHKRSCAWKDCRCPCCLLVVERQRVMAAQ	1.5e-18
Hymenoptera	*Orussus abietinus*	GAUJ01091006.1_5/57-102	-SPKCARCRNHGIISSLKGHKRSCAWKDCRCPCCLLVVERQRVMAAQ	1.7e-18
Hymenoptera	*Tenthredo koehleri*	GAWW01088411.1_2/215-260	-SPKCARCRNHGVISGLKGHKRSCAWKDCRCPCCLLVVERQRVMAAQ	3.6e-18
Hymenoptera	*Chyphotes mellipes*	GAXL01043535.1_1/29-74	-SPKCARCRNHGVISGLKGHKKSCAWKDCRCPCCLLVVERQRVMAAQ	4.9e-18
Hymenoptera	*Pseudomasaris vespoides*	GAXQ01011632.1_5/152-197	-SPKCARCRNHGVISGLKGHKRSCAWKDCRCPCCLLVVERQRVMAAQ	5.3e-18
Hymenoptera	*Pseudomasaris vespoides*	GAXQ01011634.1_5/152-197	-SPKCARCRNHGVISGLKGHKRSCAWKDCRCPCCLLVVERQRVMAAQ	5.5e-18
Hymenoptera	*Pseudomasaris vespoides*	GAXQ01011633.1_6/152-197	-SPKCARCRNHGVISGLKGHKRSCAWKDCRCPCCLLVVERQRVMAAQ	8.3e-18
Hymenoptera	*Pseudomasaris vespoide*	GAXQ01011635.1_6/152-197	-SPKCARCRNHGVISGLKGHKRSCAWKDCRCPCCLLVVERQRVMAAQ	8.5e-18
Hymenoptera	*Cotesia vestalis*	GAKG01010867.1_5/96-130	-RPKCARCRNHGLISWLRGHKRECRYKECLCPKCSL-----------	1.3e-13
Isoptera	*Mastotermes darwiniensis*	GAZE01022571.1_3/6-51	-TPKCARCRNHGVISCLKGHKRLCRWRECQCPNCQLVVERQRVMAAQ	3.7e-20
Isoptera	*Prorhinotermes simplex*	GASE01241946.1_5/28-73	-TPKCARCRNHGVISCLKGHKRLCRWRECQCPNCQLVVERQRVMAAQ	5.9e-20
Isoptera	*Nasutitermes takasagoensis*	G5ZWOJF02FLJ2Z_4/133-171	TTPKCARCRIHKELTPVKGHKRDCPHRECHCRDCYVVAKGGMYRALQ	6.9e-08*
Lepidoptera	*Spodoptera exigua*	GARL01010958.1_6/32-77	-VPKCARCRNHGLISSLRGHKKACAYRLCQCPKCGLIKERQRIMAAQ	3.8e-20
Lepidoptera	*Polyommatus icarus*	GAST01022246.1_6/28-73	-TPKCARCRNHGVISCLKGHKRLCRWRDCRCPSCLLVLERQRVMAAQ	1.6e-20
Lepidoptera	*Athetis lepigone*	GARB01040060.1_3/51-96	-VPKCARCRNHGLISSLRGHKKACAYRLCQCPKCGLIKERQRIMAAQ	2.4e-19
Lepidoptera	*Yponomeuta evonymellus*	GASG01107059.1_5/89-129	-TPKCARCRNHGVISCLKGHKRLCRWRDCRCPGCLLVLERQR-----	3.2e-16
Mantodea	*Metallyticus splendidus*	GATB01281539.1_1/31-76	-TPKCARCRNHGVISCLKGHKRLCRWRECRCPNCQLVVERQRVMAAQ	1.7e-20
Mecoptera	*Nannochorista philpotti*	GADB01005720.1_1/40-85	-TPKCARCRNHNVITDVKGHKKVCGWRNCRCPRCRLVDERQRVMAAQ	6.9e-19
Mecoptera	*Nannochorista philpotti*	GADB01001122.1_6/166-211	-TPKCARCRNHGVISCLKGHKKLCRWRECQCPNCQLVVERQRVMAAQ	2.8e-19
Mecoptera	*Panorpa vulgaris*	GAUH01056862.1_5/41-86	-VPKCARCRNHGVISGLRGHKKECLYKGCLCAKCNLIKERQRIMAAQ	1.7e-18
Neuroptera	*Conwentzia psociformis*	GAYH01081098.1_6/60-105	-VPKCARCRNHGIISSLRGHKKICAYKSCRCAKCNLIHERQRIMAAQ	7.6e-20
Neuroptera	*Osmylus fulvicephalus*	GAYC01008034.1_6/33-78	-TPKCARCRNHGIISCLKGHKRLCPWKECKCNNCQLVLQRQKIMAAQ	2.7e-18
Orthoptera	*Tetrix subulata*	GASQ01096846.1_1/5-50	-TPKCARCRNHGVISSLKGHKRLCRWRECSCASCQLVVERQRVMAAQ	3.3e-21
Orthoptera	*Ceuthophilus sp. AD-2013*	GAUX01271954.1_3/33-78	-TPKCARCRNHGVISCLKGHKRLCRWRECQCPNCQLVVERQRVMAAQ	3.7e-20
Orthoptera	*Prosarthria teretrirostris*	GAZT01143051.1_3/24-69	-TPKCARCRHHGVISSLKGHKRLCRWRECVCASCQLVVERQRVMAAQ	4e-19
Orthoptera	*Gryllotalpa sp. AD-2013*	GAWZ01138841.1_4/2-47	-TPKCARCRNHGVISCLKGHKRLCRWRE-QCPYCQLVVERQRVMAAQ	1.8e-14
Phasmatodea	*Timema cristinae*	GAVX01115626.1_6/41-86	-TPKCARCRNHGVISCLKDHKRLCRWRECQCPNCQLVVERQRVMAAQ	6.4e-20
Phthiraptera	*Menopon gallinae*	GAWR01095172.1_5/146-191	-KPKCARCRNHGVISWLKGHKRECRYKDCTCARCILIAERQKVMAKQ	8.5e-20
Phthiraptera	*Menopon gallinae*	GAWR01093074.1_6/87-132	-TPKCARCRNHGVISCLKGHKRLCRWRECQCPNCQLVVGRQKVMAAQ	5.7e-19
Plecoptera	*Leuctra sp. AD-2013*	GAUF01064618.1_6/8-53	-TPKCARCRNHGVISCLKGHKRLCRWRECKCPNCQLVVERQRVMAAQ	9.3e-21
Psocodea (Psocoptera)	*Liposcelis bostrychophila*	GAYV01083169.1_5/23-68	-TPKCARCRNHGVISCLKGHKKLCRWRECQCPNCQLVVERQRVMAAQ	1.5e-20
Raphidioptera	*Inocellia crassicornis*	GAZH01032725.1_5/40-78	-IPKCARCRNHGMISSLRGHKKLCIYKNCRCAKCNLIKER-------	9.8e-16
Siphonaptera	*Ceratophyllus gallinae*	GAWK01044190.1_6/53-98	-TPKCARCRNHGVISCLKGHKKLCRWRECQCPNCQLVVERQRVMAAQ	3.8e-20
Siphonostomatoida	*Caligus rogercresseyi*	GAZX01023775.1_4/32-77	-RPKCARCRNHGVISWLKGHKRHCAYKDCSCCKCNLIAERQRVMAAQ	5.8e-20
Thysanoptera	*Thrips palmi*	GAXC01041671.1_5/35-80	-TPKCARCRNHGVISCLKGHKKLCRWRECRCAACLLVVERQRVMAAQ	2.1e-20
Trichoptera	*Rhyacophila fasciata*	GAXX01069925.1_6/105-138	-EPKCASCKNHGVISDLKGHKRFCEWRDCECVKCR------------	4.1e-12
Zygentoma	*Occasjapyx japonicas*	GAXJ01053929.1_3/25-69	--PKCARCRNHGMISWLKGHKRHCRFKDCICAKCNLIAERQRVMAAQ	3.4e-21
Zygentoma	*Tricholepidion gertschi*	GASO01237223.1_5/12-57	-TPKCARCRNHGVISCLKGHKKLCRWRECQCPNCLLVVERQRVMAAQ	7.4e-20
Zygentoma	*Occasjapyx japonicas*	GAXJ01108043.1_3/66-111	-TPKCARCRNHGVISCLKGHKKLCRWRECQCPNCLLVVERQRVMAAQ	1.3e-19
Zoraptera	*Zorotypus caudelli*	GAYA01373377.1_2/34-79	-TPKCARCRNHGVISCLKGHKKMCRWKECECSNCQLVVERQRIMAAQ	5e-20

Figure S4

Order	Species	NCBI accession	Sequence	HMM e-val
Protura	*Acerentomon sp. AD2013*	GAXE01016537_1	AATLIFQQRLEHLLNKFKLPVESLPAIYAVLKDAKGCVEEASERLMAAYEELHEISVRLGLL	9.8e-14
Collembola	*Anurida maritima*	GAUE01011497_6	MSVDGMKESLHALLEMFRFPLESLPLVYRILKDYRADFRLASSKIMEAQTELRSLALLEAAR	2.7e-17
Collembola	*Pogonognathellus sp. AD-2013*	GATD01095902_2	GAEPLVDGMKESLQTLMDMFPNPLPLVYLILKDSRADFKLASTRILEAQAELRSIALREAAR	7.8e-11
Collembola	*Orchesella cincta*	GAMM01004046_1	TAIDEMKESVKALMEMFRLPSETDFLIYRILKDYRGDFKQASAKIFEAQCELRSWELLREAA	8.6e-12
Diplura	*Occasjapyx japonicus*	GAXJ01108506_1	DNVEEVNQALKVILKLFHLPDESCHLLWVILKDAKGDLMLASSRIMEAQSELRSMAFQDVTR	1.7e-15
Archaeognatha	*Meinertellus cundinamarcensis*	GAUG01186505_6	ENVENVKESVYGLMDRFRLPSECLPLLYVILKDARFDIKEAANRIVDAQADLHAVTLREAQY	1.6e-20
Archaeognatha	*Lepismachilis y-signata*	Contig_336923[1]	VSLETLVENCNKLLEKFHYSWEMMPLVLVILNYAGSDLDEASRKIDEGQWRSQTFCCAAN*-	5.6e-27*
Zygentoma	*Tricholepidion gertschi*	GASO01035827_5	EEGDVMMESLHALLEMFQLPLETLPLIYVVLKDARSDVKEASNRIIEAQSELRSIF------	3.3e-23
Zygentoma	*Thermobia domestica*	GASN01345779_5	ENVELLKDSLHALLDMFRLPLETLPLIYVVLKDARSDVKEASNRIMEGVYQCMNQGIIAPIG	2.4e-22
Odonata	*Calopteryx splendens*	GAYM01004193_6	DNMNFIKDAILALLQMFRLPVETLPLIYAILQGARCDVKEATNQIQNAQEHLRAMALRMMYP	1.1e-19
Ephemeroptera	*Isonychia bicolor*	GAXA01058460_2	DNIDSLRDSIQTLLDTFRLPMETLPLVYVVLKVSRSDVQEAFNRISEGGYHMTSSDVPVAAA	1.2e-20
Plecoptera	*Leuctra sp. AD2013*	GAUF01067748_5	DGHEVPFESIRKLLERFRLPSEAQPLLLAILKNSDYDSNEASKQIEIGGYNSSHPQGKAISV	2.0e-14
Plecoptera	*Perla marginata*	GATV01133007_4	PYPDVHLGSIAKLIDMCHLSPETQPLILAILKLSKFDVKEAYKQIMAGGYFYNKNCTSN---	3.6e-10
Mantophasmatodea	*Tanzaniophasma sp. AD2013*	GAXB01144312_2	ENMDGLREAIHTLLKIFQLPLETLPLLYVVLKDARSDFTEAYNRIVEARNELETLARREAAC	6.6e-20
Grylloblattodea	*Galloisiana yuasai*	GAWN01150275_2	DHGKLLWESIQALREMFHLSQDSVPLIFLVLNFSHFDVMEASIRIKEGMFVIIEYLVGTCIV	2.3e-15
Phasmatodea	*Aretaon asperrimus*	GAWC01030481_5	VSVEAMMDGVYTLLHMFHYHVEMLPLLLVVLSDAHCDVSEAYNRILQVLGNHLDSVLPVTSS	4.3e-18
Phasmatodea	*Ramulus artemis*	GAWE01101272_2	VSVEAMMDGVYTLLHMFHYHVEMLPLLLVVLSDAHCDVTEAYNRILQGECAAA---------	1.5e-18
Blattodea	*Blaberus atropos*	GAYD01026473_5	ESLEVTLQSMHYLMNTFRMPLEALPLVYVVLQLSHSDVSEATARIIKGSYST----------	6.0e-16
Blattodea	*Periplaneta americana*	GAWS01246226_6	----------QMLLEMFRFPPVALPLIYVVLQVSQSDVNVAYNRIIQAQEQLRSMALREAAR	1.6e-15
Blattodea	*Blatella germanica* genome	JPZV01136787_1	ENQEVPLQSMQYLMDFFRMPMEALPLIYVVLQLSHSDVKEAATRIIKGSYSNVIFHI*----	6.3e-06*
Thysanoptera	*Frankliniella occidentalis*	GAXD01013559_1	ENGRVHRESIQRLVEKFNFPPVALPLIYVVLKDHGSDYEEVKN-------------------	1.1e-12
Thysanoptera	*Frankliniella cephalica*	GAYE01005232_5	ENGRVHRESIQRLVEKFNFPPVALPLIYVVLKDHGSDYEEVKNLLLEAASELDETSYNQERR	6.3e-16
Thysanoptera	*Thrips palmi*	GAXC01002564_6	ENGRVHRESIQRLVEKFNFPPVALPLIYVVLKDHGSDYEEVKNLLLEAASELDQSGFNQERG	1.4e-14
Thysanoptera	*Gynaikothrips ficorum*	GAXG01131335_3	-NERVPWQSTMRLLQMFNFPENALPLIYVVLADHKADEDKVMALLEDGEF*-----------	3.1e-07
Megaloptera	*Sialis lutaria*	GABK01005713_2	HSLDVVLERCTRLLDTFMYPWEMMPLMYVILKYAGGNIDEAIRSIREGKRVVNEYSRIHNLN	8.4e-25
Megaloptera	*Corydalinae sp. KMRSPBM-2012*	GADH01041729_2	-NIELILECCHRLLDTFLYPWELMPLMYVILKYAGGNLDEAIRSIREGKRVVNEYSRTHNLN	2.1e-24
Strepsiptera	*Mengenilla moldrzyki*	JP085896_4	NETERILDNSKKLLETFKFHSEMMPLMYVILKDARTDIRKATRRIDEGWQVVNEYSIKNNLN	5.7e-22*
Strepsiptera	*Stylops melittae*	GAZM01003845_3	--------FXQKLMEIYKYPYEMMPLMYAILKSVNVDFIEAQTRIDEGHQVVSDFLRKHNLN	3.0e-16
Coleoptera	*Meloe violaceus*	GATA01002860_6	RGAENLLEFCQRLKDKFQLSWKMISLVDVILKYAKEDQEEAWRQIDEAFLEIRALAAVEAAR	2.3e-08
Coleoptera	*Meloe violaceus*	GATA01002861_6	RGAENLLEFCQRLKDKFQLSWKMISLVDVILKYAKEDQEEAWRQIDEAFLEIRALAAVEAAR	2.4e-08
Coleoptera	*Dendroctonus ponderosae*	GAFX01005311_3	DRSMDLLEDCHKLLEKFHYPYEMMPLMYAILKDARADLDEASRRIDEGKQVVNEYSRTHNLN	1.9e-25
Coleoptera	*Onthophagus nigriventris*	GAQW01004722_2	IGQKDLIQESLQLLEKFRYSWEMMPLIYAIVKDTPD-LEEASKRIDEGKDAEQLVDFLIK*-	3.1e-14
Coleoptera	*Onthophagus nigriventris*	GAQW01004722_1	*RR*TTSGFFNKIKDRFHLSWKMISLIHVILKNAKDDQEKAFRQIDEAFLEVQALAKYYPTP	5.9e-05
Trichoptera	*Hydroptila sp. AD2013*	GAVM01009688_4	ALMESILENCYILLERFNYSFEMMPLIYAILKATHIDIDEASRRIDEGQTFYLYVKDVMTAA	1.3e-19
Trichoptera	*Platycentropus radiatus*	GASS01085500_2	ASLETLLENCHKLLEKFHYSWEMMPLMYAILKDAHSDLEEASRRIDEGKWVINEYARQNNLN	9.9e-29
Lepidoptera	*Dyseriocrania subpurpurella*	GASY01001893_6	TSLETLVENCQKLLKKFHFSWEMMPLVYAIVKHAQSNLDEASQRIEEALSTSIGHYERPLTT	2.2e-16
Lepidoptera	*Dyseriocrania subpurpurella*	GASY01001894_6	TSLETLVENCQKLLKKFHFSWEMMPLVYAIVKHAQSNLDEASQRIEEGYHFSHYYHYTLLRE	7.1e-18
Lepidoptera	*Polyommatus icarus*	GAST01019581_4	VSLESLVENCHKLLEKFHYSWEMMPLVLVILNYAGSNIDEASRKIDEDVKHVVCHES-----	3.4e-19
Lepidoptera	*Yponomeuta evonymellus*	GASG01061525_3	-----LVENCNKLLEKFHYSWEMMPLVLVILNYAGSDLDEASRKIDEGKMRVDEYARKHNLN	1.4e-22
Lepidoptera	*Empusa pennata*	GAWT01314774_6	ENIAVSRESILELLDRFRLPWETLPLIYVVLDDARCDVNEASKRILKGCYAKANDKVIVNAI	2.1e-17
Siphonaptera	*Ctenocephalides felis*	GAYP01008256_1	EKVKALLEESQQIVEQLKYPFEMLPFVYAILGLSGG-ISEAIKNIHNGQLVVSEYSRMNNLN	1.6e-15
Mecoptera	*Bittacus pilicornis*	GATH01002938_5	SEVNLLLDHCQKILEKFNYPFEMMPLIYAILKDAGD-IEEASRRIDEGFLVYERLREEIRIM	1.1e-20
Mecoptera	*Boreus hyemalis*	GAYK01015512_3	YSDKVVFDLSQKLLEKFNYPFEMMPLIYANLKYAGS-IDE----------------------	3.6e-10
Mecoptera	*Boreus hyemalis*	GAYK01017535_4	YFDKVVFDLSFKLLKKFNCPIEMMPLIYANLRYADN-IDEASRRIEEDILRITYGRYHLVNL	7.4e-10
Diptera	*Bombylius major*	GATI01007471_5	QKAERLLEYCQKLLEKFRYSWKMMPVMYAILKDARADVEEASRRIDEAIRVVRPLTLHLRYP	2.0e-20
Diptera	*Bibio marci*	GATJ01028801_1	--VDVLLECSTKLLELFQYPWEMLPLMYVIVKDAGLNLEEAVRRIVEANNDIRALYFWKAVR	1.8e-19
Diptera	*Bibio marci*	GATJ01051986_1	LDDTAVVDHCQTILKRYDYPGEMLPLIYAIVQYCPN-LEEAIRRIDEGYRFYLLYTKLNSQV	3.7e-11

Figure S5

Atelura formicaria	GAYJ01004010*	ARERMLYESLVMMRQAFPVGEEAMPLLLCILKHSRS-VQEASLKIYQGHYDLNAKGFVGLDG	Zygentoma
Thermobia domestica	GASN01031735*	ARERMLYESLLVMRQAFPVGEEAMALLLCILKHSRS-VQEASWKIYQGTYDLTSRGFVGLDN	Zygentoma
Tricholepidion gertschi	GASO01256612*	EREQMLYESLMVMRQAFPVGEEAMPLLLCILKHSRS-VQEASWKICQGTRDLSARGIIGIEN	Zygentoma
Ephemera danica	GAUK01006643*	TRERMLYESLMQLRQLYPVPEASLPLLLCILKSSRS-IDEASAKIVQGTHELSTRGFPGIEA	Ephemeroptera
Isonychia bicolor	GAXA01111429*	TRERMLYESLLELRQSFPVPEAGLPLLLCILKSSRS-VHEASAKIVQGTHDLTTSGLLGLDG	Ephemeroptera
Baetis sp. AD-2013	GATU01002143*	QRKNLMYSSLLELRSHHPVPDAALPLLLCILNISNS-VKEASEKIQKGLQELSLAAASSNEL	Ephemeroptera
Atelura formicaria	FCD0KP1ACXX_7_2202_17946_4233_6[1]	------KESLQTLLDMFRLPMETLPLIYVVLKDARSDVKEASNRIIEGGYWIMTQMAA----	Zygentoma
Thermobia domestica	GASN01345779	ENVELLKDSLHALLDMFRLPLETLPLIYVVLKDARSDVKEASNRIMEGVYQCMNQGIIAPIG	Zygentoma
Tricholepidion gertschi	GASO01035827	EEGDVMMESLHALLEMFQLPLETLPLIYVVLKDARSDVKEASNRIIEAQSELRSIF------	Zygentoma
Isonychia bicolor	GAXA01058460	DNIDSLRDSIQTLLDTFRLPMETLPLVYVVLKVSRSDVQEAFNRISEGGYHMTSSDVPVAAA	Ephemeroptera

* — high-confidence EST contig

[1] — transcriptome sequencing read obtained from SRA accession SRR921568

Figure S6

```
>Sipyloidea_sipylus_contig22122
CTGTCCCGCGGAAGAAGGGGAGGGCCCCGGCTTGGGGCTTGCTTGCCTGCCACTTACTTGCACTCGCGGCGGGCAGTGAGTGCCGCGCGA
ACAAGAGTGCGTCACAAAACACCCGGTTAACCTCAAATCAACAAAACACAGCTTTAGCCGCGCGCGCATGTCGGACACCGACACCAGCAC
GGACGCGACAGCCGCCGCCGCCGCCCCCACGTGCTCGTCATCGTCGGCGGCGGTGGCCGCCAGCAGCTCGCAGAACCCGCGCACGCCGCC
GAACTGCGCTCGTTGCCGCAACCACCGTCTCAAGATCGCCTTGAAAGGCCACAAGAGATACTGCAAATACCGCTACTGCACTTGCAGCAA
GTGCCGGCTGACGGCGGAGCGACAACGCGTCATGGCGCTGCAGACGGCGTTGCGGCGGGCACAAGCACAGGACGAGAGGTACCTGGCGCA
GCAGGTGGCAGCCGTCACCGCCTCCGGCGTATCACCTCCACCGCCGCCGTTATCCTTGCATGCTGCTGCAGGTACGTGCTCGCCGCCGCC
GCTGCAGCCTACGTCGCTCGCCCCCGCTCGCTCGGCCGACGGCAGCTGTGACTCGTCGTCATCGTCTCCGTGCTCGGCTAGGGTAATCTC
CGTGCCGGCGCCTCGTAAAGCGATGGCACCCGTGCACCCACCTGACATCGCCGCCTCCACGGGGATGAGCGTGGATGCCATGATGGACGG
AGTTTACACTTTATTGCACATGTTCCACTATCATGTGGAGATGCTGCCCTTACTATTGGTCGTACTCAGTGACGCACACTGTGACGTCAC
AGAAGCCTATAACCGCATCCTGCAGGAAACCCGGCCTCGGAAATTCGGGAATGCTAAGCTATGGTTGTCAGTATCATTCGTACGTGTTTG
CTGAAGTAGATATGGCGACTCCAAACTGCTGATGAAGACTGTCATCGGCTGGGAACAACTCAACTACGGACCAGATTGGCGCAGCTTCTA
GAATGCCAAGGAAGAGGTGATAGCGATGGGGCGGCGGGAGGCGGCGCGATTGATGCAGTACCATCCGGCGGCGGCGGCGACCTACGGTGG
CTGCTACGGATCCGCAGCGCCCGCATACCTACCGGGGATGACCGCTAACGGGGCAATCTACCCCACGCCGCCTCCAAGTCTCATGTTCTC
ACCTCCTAGCGCCGCCGCC

>Lepismachilis_y-signata_contig_383881
CGACCCCGGCGCCTCCAGCTCAAGCTCGGCCGTGCCGCGTGCGCCGCCCAACTGCGCGCTGCCGCAACCACCGGCTCAAGGTCGAGCT
GAAGGGTCACAAGCGCTACTGCAAGTACCGCTACTGCAACTGCGAGAAGTGCCGGCTGACGGCGGACCGGCAGCGTGTGATGGCGCTGCA
GACGGCGCTGCGGCGGGCGCAGGCGCAGGACGAGGCGCGCGCGCAACAACGGCCATCCGCCGCCGGGCGTGGAGCTGGAATGCCCGGA
GCCGCCGGTGGTGAAGGCGCCGCGCAGCCCGGTGATCCCGCCGCGGTCCGTGGGCTCTACCAGCGGCGAGTCGGTGCCGGGGTCGCCGGT
GGTGTCTCCGTACGCGGCGCCGCCGCCCTCTGCGCCGCCGCCAACCATGCCGCCGCTTCTCCCGCCACAACAGCCTGT

>Lepismachilis_y-signata_contig_336923
CCTTCATCTATCTTCCGTGACGCCTCATCCAGGTCGCTCCCCGCATAGTTGAGGATCACCAGGACTAAGGGCATCATCTCCCAGGAGTAG
TGGAACTTCTCCAGGAGTTTGTTGCAGTTTTCAACCAGCGTCTCGAGGGATACAGGCTGTTGTGGCGGGGAAGCGGGGGCACGGGTGGG
GGGGGGCAGAGGGCGGGGGGGCCGCGGACGGGGGCACCACCCGCG
```

Supplementary Figure Captions

Supplementary Figure S1. High-confidence *doublesex* transcripts reported in this study. Highly conserved amino acid positions are in red text. The conserved Lysine residue characteristic of the *dsx* DM domain (OD1, see Results and Discussion) is highlighted in yellow. Deviations from the canonical Cys-His zinc finger motif are highlighted in blue. Also included are OD2-encoding singleton domains recovered for taxa that possessed a high-confidence EST, to illustrate evidence for putative *dsx* paralogy.

Supplementary Figure S2. All type-A OD1-encoding singletons recovered in our analyses. Highly conserved amino acid positions are in red text. The conserved Lysine residue characteristic of the Type-A *dsx* DM domain (OD1, see Results and Discussion) is highlighted in yellow. E-values were calculated against our profile HMM using HMMer v3.1; asterisks indicate e-value was calculated via NCBI Conserved Domain Database search.

Supplementary Figure S3. All type-B OD1-encoding singletons recovered in our analyses. Highly conserved amino acid positions are in red text. The conserved Isoleucine residue characteristic of the Type-B *dsx*-lik*e* DM domain (OD1, see Results and Discussion) is highlighted in yellow. E-values were calculated against our profile HMM using HMMer v3.1; asterisks indicate e-value was calculated via NCBI Conserved Domain Database search.

Supplementary Figure S4. All dimerization (OD2) domain-encoding singletons recovered in our analyses. Highly conserved amino acid positions are in red text. E-values were calculated against our profile HMM using HMMer v3.1; asterisks indicate e-value was calculated via NCBI Conserved Domain Database search.

Supplementary Figure S5. Alignment of both putative *doublesex* transcripts from each of the Zygentoma and Ephemeroptera species reported in this study. The high-confidence ESTs (black text) share similarity with each other, while the alternate motif (red text) segregates.

Supplementary Figure S6. De-novo assembled contigs referenced in this study.
